# The anti-estrogen receptor drug, tamoxifen, is selectively Lethal to P-glycoprotein-expressing Multidrug resistant tumor cells

**DOI:** 10.1186/s12885-022-10474-x

**Published:** 2023-01-06

**Authors:** Rowa Bakadlag, Georgia Limniatis, Gabriel Georges, Elias Georges

**Affiliations:** 1 Institute of Parasitology, Macdonald Campus, McGill University, Ste. Anne de Bellevue, Québec, H9X-3V9 Canada; 2 Department of Cardiac Surgery, Quebec Heart & Lung Institute, Université Laval, Québec, Canada

**Keywords:** Collateral sensitivity, P-glycoprotein, ABCB1-knockout, Oxidative stress, Rotenone and Tamoxifen

## Abstract

**Background:**

P-glycoprotein (P-gp), a member of the ATP Binding Cassette B1 subfamily (ABCB1), confers resistance to clinically relevant anticancer drugs and targeted chemotherapeutics. However, paradoxically P-glycoprotein overexpressing drug resistant cells are “collaterally sensitive” to non-toxic drugs that stimulate its ATPase activity.

**Methods:**

Cell viability assays were used to determine the effect of low concentrations of tamoxifen on the proliferation of multidrug resistant cells (CHO^R^C5 and MDA-Doxo^400^), expressing P-gp, their parental cell lines (AuxB1 and MDA-MB-231) or P-gp-CRISPR knockout clones of AuxB1 and CHO^R^C5 cells. Western blot analysis was used to estimate P-gp expression in different cell lines. Apoptosis of tamoxifen-induced cell death was estimated by flow cytometry using Annexin-V-FITC stained cells. Oxidative stress of tamoxifen treated cells was determined by measuring levels of reactive oxygen species and reduced thiols using cell-permeant 2',7'-dichlorodihydrofluorescein diacetate (H2DCFDA) and 5,5-dithio-bis-(2-nitrobenzoic acid) DTNB, respectively.

**Results:**

In this report, we show that P-gp-expressing drug resistant cells (CHO^R^C5 and MDA-Doxo^400^) are collaterally sensitive to the anti-estrogen tamoxifen or its metabolite (4-hydroxy-tamoxifen). Moreover, P-gp-knockout clones of CHO^R^C5 cells display complete reversal of collateral sensitivity to tamoxifen. Drug resistant cells exposed to low concentrations of tamoxifen show significant rise in reactive oxygen species, drop of reduced cellular thiols and increased apoptosis. Consistent with the latter, CHO^R^C5 cells expressing high levels of human Bcl-2 (CHO^R^C5-Bcl-2) show significant resistance to tamoxifen. In addition, the presence of the antioxidant N-acetylcysteine or P-gp ATPase inhibitor, PSC-833, reverse the collateral sensitivity of resistant cells to tamoxifen. By contrast, the presence of rotenone (specific inhibitor of mitochondria complex I) synergizes with tamoxifen.

**Conclusion:**

This study demonstrates the use of tamoxifen as collateral sensitivity drug that can preferentially target multidrug resistant cells expressing P-gp at clinically achievable concentrations. Given the widespread use of tamoxifen in the treatment of estrogen receptor-positive breast cancers, this property of tamoxifen may have clinical applications in treatment of P-gp-positive drug resistant breast tumors.

**Graphical Abstract:**

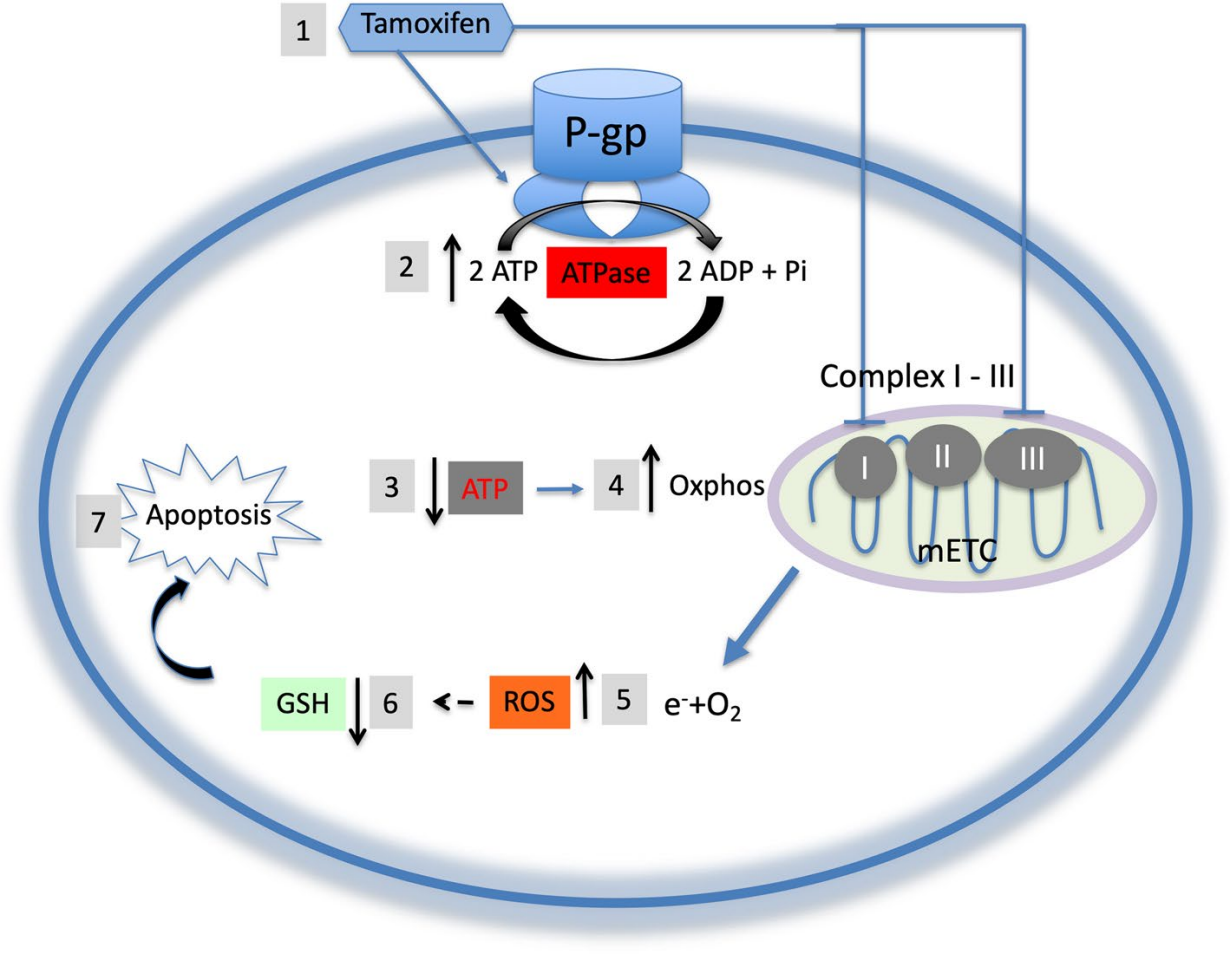

**Supplementary Information:**

The online version contains supplementary material available at 10.1186/s12885-022-10474-x.

## Introduction

Clinical drug resistance, intrinsic or acquired, remains an obstacle in the treatment of cancer patients with chemotherapeutic drugs [1–3]. P-glycoprotein (P-gp or ABCB1), a member of a large family of ATP Binding Cassette (ABC) transporters, mediates the active efflux of anticancer drugs [4]. P-gp is expressed on the cell surface in normal tissues and organs, where it mediates the secretion of xenobiotic and normal cell metabolites [5]. Increased P-gp expression in tumors post chemotherapeutic treatment have been shown in several cancers [6]. Moreover, its expression in tumors from breast, neuroblastoma and lung cancers have been associated with poor prognosis and patients outcome [7, 8]. Clinical trials using increasingly more potent and specific inhibitors of P-gp drug efflux function (1^st^ to 3^rd^ generation MDR-reversing drugs) have been largely unsuccessful due to unacceptable toxicity, when combined with cytotoxic anti-cancer drugs, likely due to altered drug pharmaco-kinetics [9, 10]. Consequently, attempts to block P-gp drug efflux function in the clinic have been discontinued despite the role of P-gp in tumor response to chemotherapeutic drugs [7, 8].

Earlier reports, using drug resistant cells expressing P-gp, were shown to be “*hypersensitive* or *collaterally sensitive*” to calcium channel blockers and other membrane active compounds [11–13]. We have recently shown that the collateral sensitivity of resistant cells to these drugs is dependent on P-gp expression and activity, whereby stimulation of its ATPase activity triggers a rise in reactive oxygen species (ROS) leading to heightened oxidative cell death of resistance cells [14–17]. Multidrug resistant cells were also shown to be collaterally sensitive to ROS generating compounds (*e.g.*, NSC-73306) that do not stimulate P-gp ATPase [18], but trigger oxidative cell death conditional on P-gp expression and basal ATPase activity. Although P-gp-dependent collateral sensitivity has been described in several tumor cell lines with intrinsic or acquired MDR phenotype [15, 19], certain MDR cell lines do not show P-gp-dependent collateral sensitivity. To evaluate the prospect of using collateral sensitivity drugs in the treatment of drug resistant cancers, it was of interest to identify non-toxic and clinically approved drugs that can trigger P-gp-dependent collateral sensitivity. Based on the working hypothesis of P-gp-dependent collateral sensitivity [14–17], we examined the ability of tamoxifen as a collateral sensitivity drug for two reasons: a) tamoxifen has been previously shown to activate P-gp ATPase at clinically achievable concentration (4–6 μM) [20, 21], and b) tamoxifen is widely used in the treatment of estrogen receptor-positive breast cancer [22]. In addition, an earlier study has shown that tamoxifen acts and an MDR-reversing drug in drug resistant P388/ADR murine leukemia cells [23], and can induce apoptosis, in both estrogen receptor-α positive and –negative breast cancer cells, by increasing intracellular ROS levels [24]. Hence, tamoxifen combines two favorable properties associated with compounds that elicit P-gp-dependent collateral sensitivity. In this study, we show that tamoxifen-induced collateral sensitivity of drug resistant cells is conditional on P-gp expression and ATPase activity. Moreover, collateral sensitivity to tamoxifen is ROS-mediated and acts synergistically with rotenone (a ROS generating drug).

## Material and methods

### Materials

Tamoxifen, 4-hydroxy-tamoxifen, Dimethylsulfoxide, 2’,7’-dichlorofluorescin diacetate, 5,5’-dithio-bis (2-nitrobenzoic acid), colchicine, valspodar (PSC-833) and Tween-20 (P9416) were all purchased from Sigma Aldrich. Lipofectamine 2000, the microBCA kit and the Pierce™ ECL Western Blotting Substrate Kit were all products of Thermo Fisher. Propidium iodide and the HRP-conjugated goat anti-mouse were purchased from Invitrogen, and the IncuCyte® is a product of Essen BioScience.

### Tissue culture and cell growth assays

Drug-sensitive (AuxB1, MDA-MB-231), -resistant (CHO^R^C5, CHO^R^C5/Bcl-2, MDA-Doxo^400^), and P-gp-knockout (AuxB1^ΔP−gp^, CHO^R^C5^ΔP−gp−A1^, CHO^R^C5^ΔP−gp−A3^) cells were grown in α-minimal essential medium (α-MEM) or Dulbecco’s modified eagle medium, supplemented with 10% fetal bovine serum (FBS; Gibco, Life technologies) at 37 °C in the presence of 5% CO_2_, without or with selective concentrations of colchicine or doxorubicin (5 μg/ml for CHO^R^C5 and 400 nM for MDA-Doxo^400^ cells; Sigma-Aldrich, Ont., CA). For cell proliferation assays, drug sensitive and resistant cells were plated in 200 μl α-MEM containing 10% FBS in 48-well plates. Cells were incubated for 24 h at 37 °C prior to the addition of 200 μl media containing colchicine, tamoxifen, hydrogen peroxide or rotenone alone and in combinations (Sigma-Aldrich, Ont., CA). Cell colonies were allowed to proliferate for 7–8 days at 37 °C without or with drugs prior to the addition of cell-staining dye, methylene blue (0.1—1% methylene blue in ethanol/H_2_O). The dye solution was removed, and plates were washed gently in cold water and air-dried. The dye was extracted from fixed and stained cells with 0.1% SDS/PBS and the absorbance quantified at 660 nm (Dynatech Laboratories, MR5000). The effects of drugs on cell proliferation were determined by comparing the absorbance of cells grown in the presence of drugs to solvent control without added drugs (100% cell survival). All graphs shown represent the mean ± SD of three independent experiments done in triplicate. To assess the combined effects of different drugs, and measure potential drug synergy on cell proliferation, the method of Chou-Talay was used [25]. Briefly, clonogenic cell proliferation assays were set up as described above using tamoxifen, rotenone, tamoxifen together with varying non-toxic concentrations of rotenone (0.4 nM or 1.3 nM) and rotenone together with varying non-toxic concentrations of tamoxifen (0.15 μM or 0.4 μM). The measured IC_50_ values of tamoxifen or rotenone for AuxB1 or CHO^R^C5 were plotted on a Cartesian plane, and a linear regression was produced using the two points. The IC_50_ value of tamoxifen together with 0.4 nM or 1.3 nM of rotenone and rotenone together with tamoxifen (0.15 μM or 0.4 μM), normalized comparatively using the solvent control were plotted on the resulting graph for each cell line. Synergy is predicted when the resulting IC_50_ values for the combined drugs fall below the line connecting the two IC_50_ points for each drug alone on the x- and y-axes.

### CRISPR/Cas9 knockout of P-gp in CHO cell lines

eSpCas9(1.1) was a gift from Feng Zhang (Addgene plasmid #71,814; http://n2t.net/addgene:71814;RRID:Addgene_71814). The following guide-RNA sequences were designed against TMD1 of *Cricetulus griseus* P-gp, forward 5’-CACCGCTTATAGTTGCCTACATTC-3’ and reverse 5’-AAACGAATGTAGGCAACTATAAGC-3’ primers. The constructs were transformed into TOP10 cells, and empty plasmid or plasmid containing the guide-RNA was isolated and transiently transfected into the AuxB1 or CHO^R^C5 cells using the Lipofectamine 2000 kit (Invitrogen). Populations of the transfected cells were grown, and knockout clones were isolated via serial dilution method. The absence of P-gp was verified by Western blot using P-gp-directed monoclonal antibodies, C219 and C494 mAbs which bind different epitopes in P-gp ATPase domain conserved in human and hamster [26].

### Apoptosis assays

For annexin-V staining of apoptotic cells, drug sensitive and resistant cells (AuxB1 and CHO^R^C5, respectively) were seeded in six-well plates at 1–2 × 10^5^ per well and incubated for 24 h prior to the addition of tamoxifen for 24 h. Cells (1 × 10^6^) were lifted and washed with cold PBS and resuspended in 100 μl binding buffer according to the manufacturer’s protocol (BD, FITC Annexin V Apoptosis Detection kit I). Briefly, apoptotic cells stained with the addition of Annexin-V-FITC solution (5 μl) and propidium iodide (5 μl), allowed to incubate for 15 min in the dark, then diluted with 400 μl of binding buffer prior to analysis by flow cytometry (BD FACSDiva). Percent apoptosis was determined by measuring the relative fluorescence in drug treated versus control untreated cells. For Hoechst dye staining of apoptotic cells, AuxB1 and CHO^R^C5 cells were incubated without and with 5 μM tamoxifen for 24 h. Hoechst 33,258 dye (1 μg/mL) was added for 10-min at 37 °C prior to observing cells under UV light to assess the percent of apoptotic cell. Photographs were taken at 2000X magnification (Nikon, Eclipse TE200, Quebec, Canada).

### ROS measurements

AuxB1 and CHO^R^C5 cells were seeded at a density of 50,000 cells/well in a clear-bottomed black-well plate and allowed to adhere for 24 h, after which the media was removed, and cells incubated with 100 μM H_2_DCFDA for 45 min at 37 °C. Wells were washed with sterile cold HBSS, followed by the addition of 100 μl of fluorobrite DMEM + 10% FBS to each well. Tamoxifen (1 μM and 5 μM) were added and allowed to incubate for another 24 h, after which the fluorescence signals were measured at 485_ex_, 527_em_ using in the H4 Synergy plate reader (BioTek Inc., USA). The fluorescence signals from cells treated with tamoxifen relative to solvent control were plotted using GraphPad Prism (GraphPad Software, version 8.0.1).

### Measurement of total reduced thiols

Cells (AuxB1 and CHO^R^C5) were seeded at 100,000 cells/well in 48-well plate and allowed to adhere for 24 h, after which increasing doses of tamoxifen were added. Following another 48-h incubation, the media was removed and 250 μl of RIPA buffer (50 mM Tris, 150 mM NaCl, 0.5% sodium deoxycholate, 1% NP-40, 0.1% SDS, pH 8) was added to each well. Plates were mixed for 20 min and 50 μl of each sample was removed for protein measurement using micro-BCA protein assay (ThermoFisher Scientific Inc. USA). To the remaining samples, 50 μl of 50 mM DTNB (5,5-dithio-bis-(2-nitrobenzoic acid) or Ellman's Reagent) was added and incubated for 30 min. The tissue culture plates were scanned at 412 nm on Synergy H4™ Hybrid Multi-Mode Microplate reader (Biotek Inc. USA). Analysis was done comparing the absorbance of each sample to the total amount of protein in the sample, yielding the relative amount of sulfhydro moieties compared to solvent control. The data were analyzed and the total amount of sulfhydro groups compared to their respective solvent control were plotted using GraphPad Prism (GraphPad Software, version 8.0.1).

### Protein extraction and immuno-detection

Total cell lysates (20 μg) from drug-sensitive and -resistant cells were resolved on 6% SDS-PAGE and transferred onto PVDF membrane. The membrane was blocked in milk in phosphate buffered saline (PBS) at 5% (w/v) and probed with P-gp specific mAb (0.1 μg/ml of C219 mAb) or anti-Bcl-2 (0.5 μg/ml; BioLegend, San Diego, USA; anti-Bcl-2 (D55G8) rabbit mAb, 1:1000 v/v (human specific), Cell Signaling Technology, Massachusetts, USA; or anti-β-Actin mAb, 0.5 μg/ml, Sigma-Aldrich, Toronto, CA) in 5% milk/PBS, followed by several washes in PBS and incubation with horseradish peroxidase (HRP)-conjugated goat anti-mouse IgG (1:5000 v/v, BioRad, Ont., CA). The reactive signals were visualized using Western Breeze Chemiluminescent Kit and captured using ECL-imager from Thermo-Fisher Scientific. Tubulin expression was detected on the same PVDF membrane using anti-α-tubulin specific monoclonal antibody (1 μg/ml Sigma-Aldrich, Ont., CA), followed by HRP-conjugated goat anti-mouse IgG (1:5000 (v/v), BioRad, Ont., CA).

### In vitro isobologram analysis

The effects of tamoxifen alone and in combination with rotenone on the proliferation of drug-sensitive (AuxB1 and MDA-MD-231) and –resistant (CHO^R^C5 and MDA-Doxo^400^) cells were assessed using the modified fixed-ratio isobologram analysis protocol. This method is usually adopted to measure the degree of chemo-sensitization of a given compound and to detect if the effect between two drugs is synergistic, additive, or antagonistic [27]. A stock of tamoxifen and rotenone combination was prepared in fixed-concentration ratios. Drug-sensitive and resistant cells were incubated with the above drug combination for 72 h, and a nonlinear regression curve was used to determine the IC_50_ values for each drug alone and in combination. Fractional inhibitory concentrations (FICs) were then calculated using the equations below, as previously described [28, 29].  

$$\begin{array}{c}\mathrm{FIC_{tam}}=\frac{\mathrm{IC_{50}}\;\mathrm{of}\;\mathrm{tamoxifen}\;\mathrm{in}\;\mathrm{combination}}{\mathrm{IC_{50}}\;\mathrm{of}\;\mathrm{tamoxifen}\;\mathrm{alone}}\\\mathrm{FIC_{rotenone}}=\frac{\mathrm{IC_{50}}\;\mathrm{of}\;\mathrm{rotenone}\;\mathrm{in}\;\mathrm{combination}}{\mathrm{IC_{50}}\;\mathrm{of}\;\mathrm{rotenone}\;\mathrm{alone}}\\\mathrm{FIC_{index}}=\mathrm{FIC_{tam}}+\mathrm{FIC_{rotenone}}\end{array}$$

The isobologram curves were constructed by plotting FIC_tam_ vs FIC_rotenone_. The drug combination effect was assessed from the graphs but also by calculation. A straight diagonal line or FIC_index_ = 1 indicates a clear additive effect between the two drugs, while a concave curve below the diagonal of the graph denotes a synergistic effect between the drugs. In addition, the synergy may be strong or moderate when the means of FIC_index_ values are respectively below 0.5 or between 0.5 and 1. A convex curve above the diagonal indicates an antagonistic effect and demonstrates an absence of synergy with the FIC_index_ > 4. An FIC_index_ between 1 and 4.0 is defined as no interaction [30].

### Statistical analysis

All graphs and statistics were performed using GraphPad prism version 6. Statistics represent the student *t* and one-way ANOVA test.

## Results

### P-glycoprotein expression and collateral sensitivity to tamoxifen

**Fig. 1:**
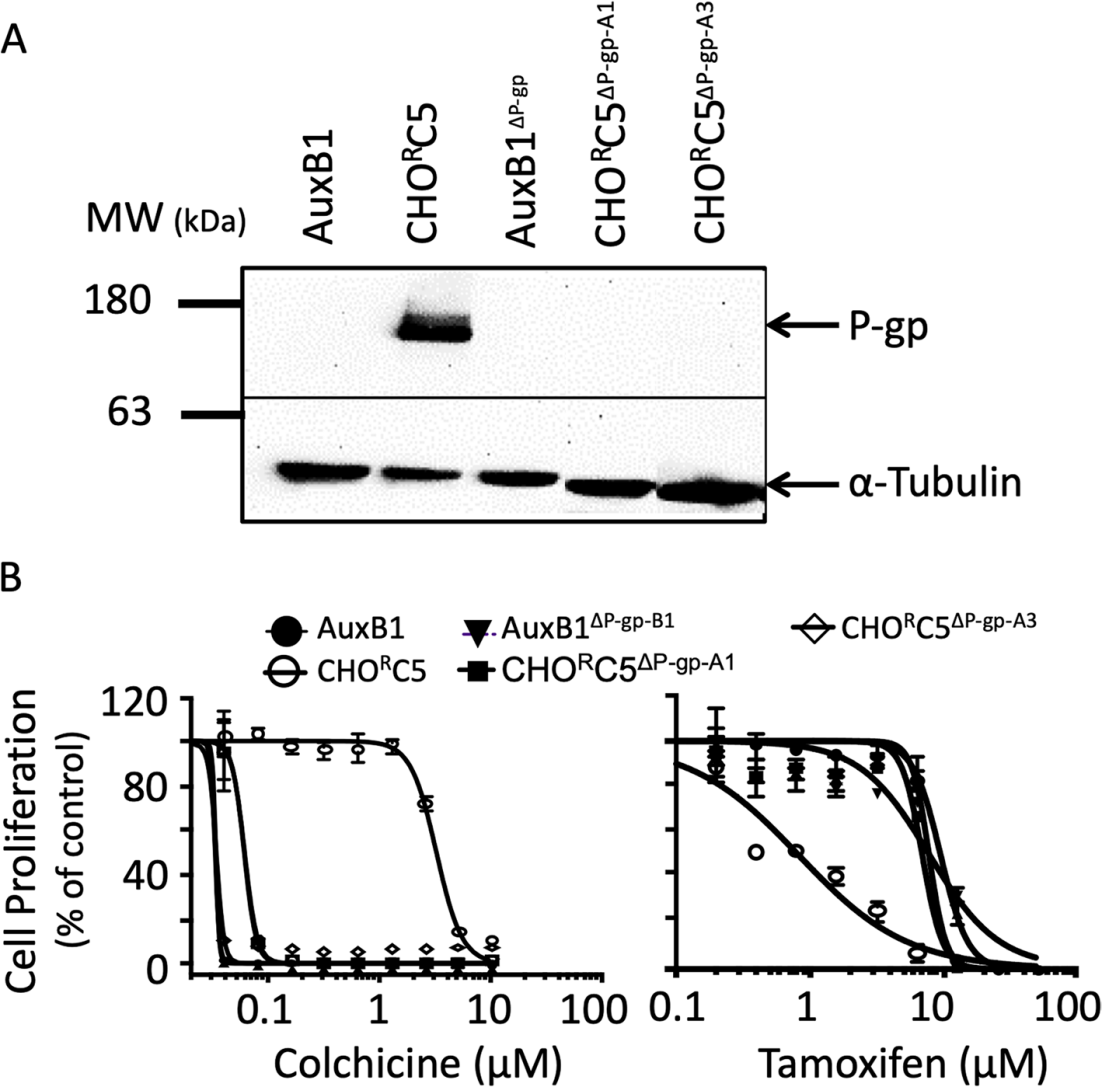
Collateral sensitivity of drug resistant cells to tamoxifen is dependent on P-gp expression- Panel (**A**) shows a Western blot analysis demonstrating the expression of P-gp in total protein extracts from wild-type Chinese hamster ovary cells (AuxB1), drug resistant selected cells (CHO^R^C5), P-gp-knockout drug sensitive cells (AuxB1^ΔP−gp^), and P-gp-knockout CHO^R^C5 (CHO^R^C5^ΔP−gp−A1^, and CHO^R^C5^ΔP−gp−A3^) cells, probed with P-gp-directed monoclonal antibody (C219 mAb) and anti-α-tubulin. α-tubulin expression is shown as a loading control. Panel (**B**) shows the proliferation of the above cell lines (AuxB1, CHO^R^C5, AuxB1^ΔP−gp^, CHO^R^C5^ΔP−gp−A1^, and CHO^R^C5^ΔP−gp−A3^) without or with increasing molar concentrations of colchicine (0–10 µM) or tamoxifen (0–50 µM). Graphs represent the mean ± SD of three independent experiments done in triplicate

**Fig. 2:**
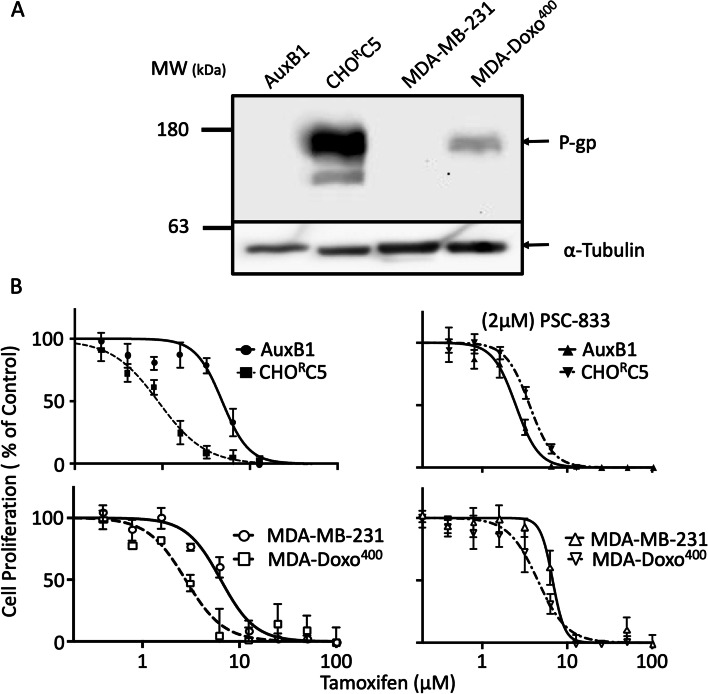
P-gp expression levels and ATPase activity modulate collateral sensitivity to tamoxifen—Panel (**A**) shows P-gp expression in total cell lysates from drug sensitive (AuxB1 and MDA-MB-231), and – resistant (CHO^R^C5, MDA-Doxo^400^) cells probed with P-gp-specific monoclonal antibody (C219 mAb) and anti-α-tubulin. Panel (**B**) shows the proliferation of the above drug sensitive and resistant cell lines (AuxB1, MDA-MB-231, CHO^R^C5, MDA-Doxo^400^) in the presence of increasing concentrations of tamoxifen, without and with 2 μM PSC-833. Graphs represent the mean ± SD of three independent experiments done in triplicate

The impact of P-gp expression on the collateral sensitivity of Chinese hamster cells to tamoxifen was assessed using drug-sensitive, drug-resistant cells and their respective P-gp-knockout cells. Figure 1A shows the results of a Western blot probed with P-gp-specific monoclonal antibody (C219) revealing the relative expression of P-gp in total cell extracts from drug sensitive Chinese hamster ovary cells (AuxB1), AuxB1 colchicine-selected drug resistant cells (CHO^R^C5), P-gp-knockout drug sensitive cells (AuxB1^ΔP−gp^) and P-gp-knockout drug resistant cells (CHO^R^C5^ΔP−gp−A1^, and CHO^R^C5^ΔP−gp−A3^). CHO^R^C5 drug resistant cells show high P-gp expression relative to drug sensitive AuxB1, or P-gp-knockout cells (AuxB1^ΔP−gp^, CHO^R^C5^ΔP−gp−A1^, and CHO^R^C5^ΔP−gp−A3^). To rule out the possibility that the observed Western blot signal for P-gp in Fig. 1A is due to cross-reactivity of C219 mAb with HER2 protein migrating with a molecular mass of ~ 185 kDa [31, 32]; a Western blot containing cell extracts as in Fig. 1A was probed with another P-gp-directed monoclonal antibody (C494 mAb) which binds to a different epitope in human and hamster P-gp than that of C219 mAb [26]. The results of this latter Western blot (supplemental Fig. 1) are consistent with those in Fig. 1A. Figure 1B shows cell proliferation graphs for the above cell lines grown in culture without and with increasing concentrations of colchicine (0–10 μM) or tamoxifen (0–50 μM), respectively. The results of Fig. 1B show the drug selected CHO^R^C5 cells to be highly resistant to colchicine and collaterally sensitive to tamoxifen, relative to their parental drug sensitive AuxB1 cells. By contrast, P-gp-knockout cells (AuxB1^ΔP−gp^, CHO^R^C5^ΔP−gp−A1^, and CHO^R^C5^ΔP−gp−A3^) were highly sensitive to colchicine and resistant to tamoxifen (Fig. 1B). These results demonstrate the dominant role of P-gp in drug resistance and collateral sensitivity phenotypes of Chinese hamster tumor cells. To determine if P-gp expression levels predicts the degree of collateral sensitivity to tamoxifen, and the role of P-gp ATPase activity, two drug resistant cell lines (e.g., CHO^R^C5 and MDA-Doxo^400^) expressing different levels of P-gp were allowed to proliferate in the presence of increasing concentrations of tamoxifen, without or with specific inhibitor of P-gp-ATPase activity (PSC-833 [33];). Figure 2A shows the relative P-gp protein expression in CHO^R^C5 and MDA-Doxo^400^ cells, relative to their respective parental drug sensitive cells (e.g., AuxB1 and MDA-MB-231). CHO^R^C5 cells show significantly higher levels of P-gp expression (Fig. 2A). It is important to note that both human and hamster P-gp contain the same epitope sequence for C219 mAb [26], hence differences in antibody antigen binding is not responsible for differences in P-gp signal in the two cell lines. Figure 2B shows the proliferation of drug sensitive and resistant cells in the presence of increasing concentrations of tamoxifen. Although, both CHO^R^C5 and MDA-Doxo^400^ are more sensitive to tamoxifen than their respective parental drug sensitive cells (AuxB1 and MDA-MB-231), CHO^R^C5 cells are more sensitive to tamoxifen than MDA-Doxo^400^ (IC_50_ values of 0.900 ± 0.0900 μM and 2.998 ± 0.0714 μM, respectively). The latter results are consistent with findings from earlier studies demonstrating a correlation between P-gp expression levels and the degree of hypersensitivity of drug resistant cells to collateral sensitivity inducing drugs [15, 16]. It is noteworthy that the observed collateral sensitivity of MDA-Doxo^400^ cells to tamoxifen is estrogen receptor-independent [34]. Moreover, the results in Fig. 2B show that the presence of 2 μM PSC-833, a specific inhibitor of P-gp ATPase [33], reverses the collateral sensitivity of CHO^R^C5 and MDA-Doxo^400^ cells to tamoxifen, with shift in IC_50_ values from 0.900 ± 0.090 and 3.582 ± 0.048 μM to 2.998 ± 0.071 and 4.336 ± 0.895 μM with PSC-833 for CHO^R^C5 and MDA-Doxo^400^, respectively. Similar results were obtained using increasing concentrations of 4-hydroxy-tamoxifen, an active metabolite of tamoxifen [35], without and with 2 μM PSC-833 (supplemental Fig. 2).

### Tamoxifen induces apoptosis in P-gp expressing cells

**Fig. 3:**
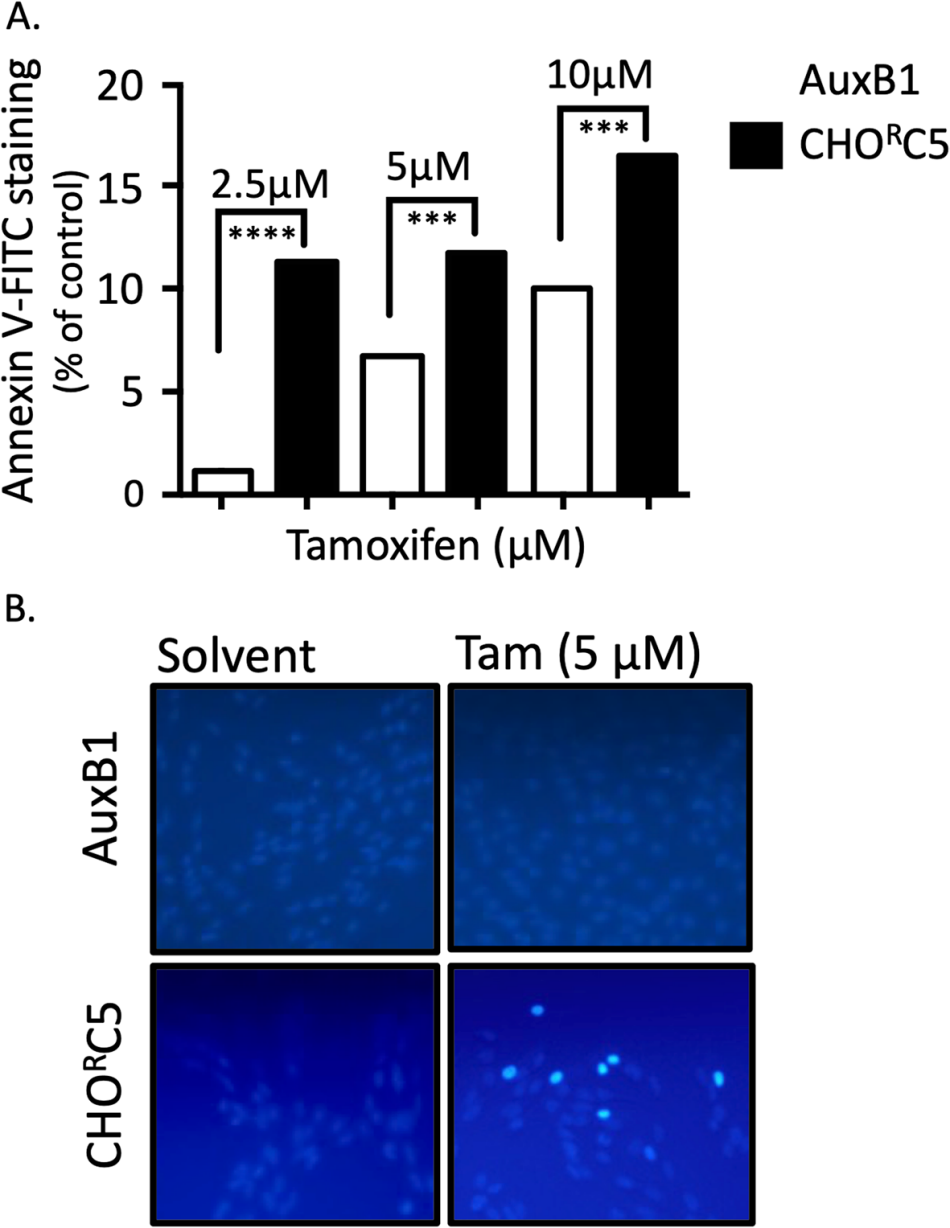
Tamoxifen promotes apoptosis in drug resistant cells—Cells were treated for 24 h with tamoxifen prior to quantification of apoptotic cells by flow cytometry or staining with Hoechst 33,258. Panel (**A**) shows the percentage of AuxB1 and CHO^R^C5 cells stained with FITC-conjugated annexin-V following 24 h treatment with tamoxifen (2.5–10 μM) relative to solvent control. (****), (***) indicates, *P* < 0.0005, statistically significant difference. Panel (**B**) shows Hoechst 33,258 stained AuxB1 and CHO^R^C5 cells following 24 h treatment with 5 μM tamoxifen versus solvent control treated cells

To determine if tamoxifen-induced collateral sensitivity of drug resistant cells is due to enhanced apoptosis, AuxB1 and CHO^R^C5 cells were treated with tamoxifen for 24 h and then examined for the presence of apoptotic cells using FITC-conjugated annexin-V and cell staining with Hoechst Dye. The results in Fig. 3A show higher percentage of drug resistant cells (CHO^R^C5) stained with Annexin V-FITC than drug sensitive (AuxB1) cells at different concentrations of tamoxifen (*i.e.*, 2.5, 5 and 10 μM). Similarly, AuxB1 and CHO^R^C5 cells treated with solvent or 5 μM tamoxifen for 24 h, then stained with Hoechst 33,258 dye and examined under UV light show the presence of apoptotic cells only among the CHO^R^C5 cells, as evident by the cells with stained nuclei (Fig. 3B), consistent with earlier findings [14, 15, 17]. Similar results were observed with MDA-MB-231 and MDA-Doxo^400^ cells treated with 5 μM tamoxifen and then stained with Hoechst 33,258 dye. Apoptotic dye-stained nuclei are observed only among MDA-Doxo^400^ cells (supplemental Fig. 3).

**Fig. 4:**
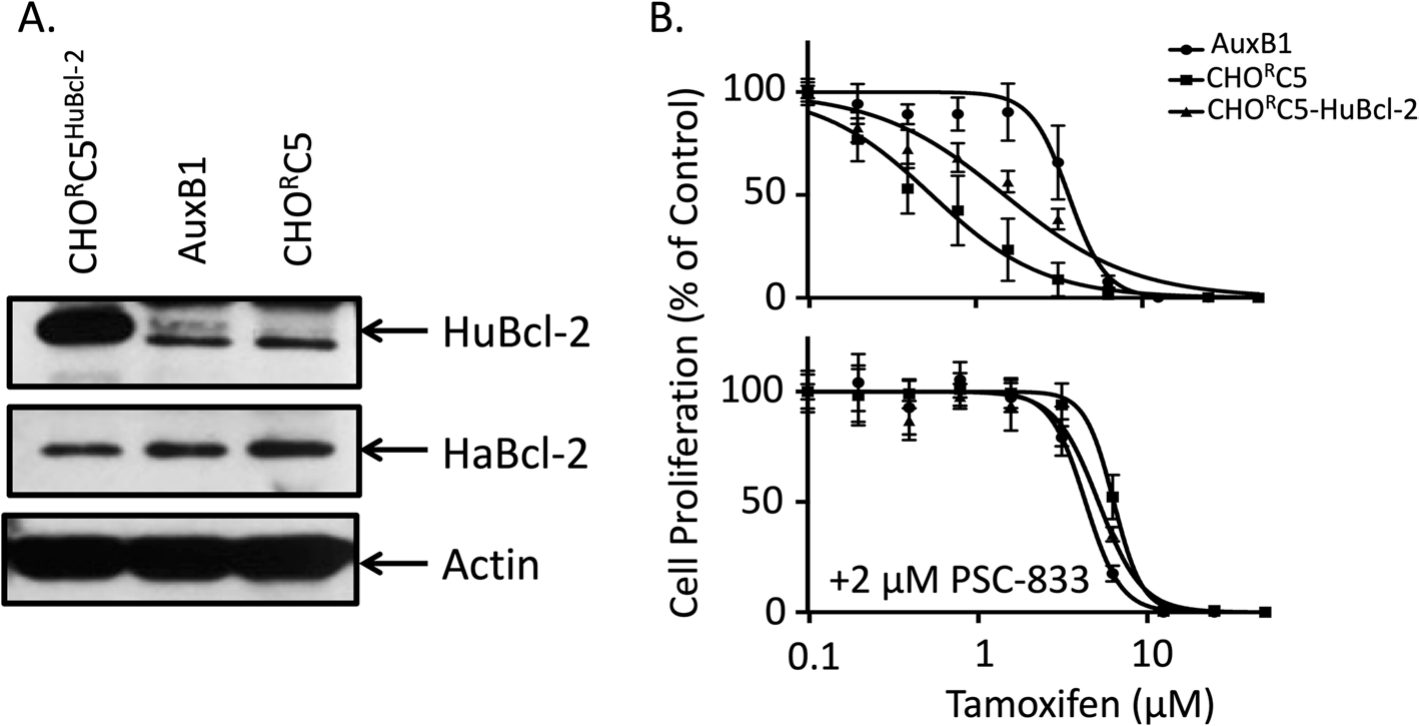
Overexpression of huBCL-2 diminishes collateral sensitivity to tamoxifen—Panel (**A**) shows a Western blot of cell extracts from AuxB1, CHO^R^C5, and CHO^R^C5 cells transfected with recombinant human Bcl-2 (CHO^R^C5^Bcl−2^) probed with anti-Bcl-2 antibody or human-specific anti-Bcl-2 rabbit mAb together with anti-β-actin mAb as loading control. Panel (**B**) shows the proliferation of AuxB1, CHO^R^C5 and CHO^R^C5^BCL2^ cell in the presence of increasing concentrations of tamoxifen without and with 2 μM PSC-833. Graphs represent the mean ± SD of three independent experiments done in triplicates

Given the results above in Fig. 3, we examined the effects of recombinant overexpression of human Bcl-2 (huBcl-2) in CHO^R^C5 (CHO^R^C5/huBcl-2) cells on their collateral sensitivity to tamoxifen. Figure 4A shows a Western blot with anti-Bcl-2 antibody and anti-β-actin mAb or human-specific anti-Bcl-2 rabbit mAb and anti-β-actin mAb to probe cells extracts from AuxB1, CHO^R^C5 and CHO^R^C5/huBcl-2. CHO^R^C5/huBcl-2 reveals high-expression level of recombinant human Bcl-2 without significantly affecting the endogenous hamster Bcl-2 expression levels (Fig. 4A). Figure 4B shows the effects of increasing tamoxifen concentrations on the *in-vitro* proliferation of AuxB1, CHO^R^C5 and CHO^R^C5/huBcl-2 cells in the absence or presence of PSC-833. These results show CHO^R^C5/Bcl-2 cells to be less sensitive to tamoxifen collateral sensitivity than CHO^R^C5 cells (IC_50_ 0.593 ± 0.28 μM versus 1.49 ± 0.23 μM, respectively), consistent with a protective effect likely due to the overexpression of recombinant huBcl-2. Moreover, the addition of PSC-833 completely reversed the sensitivity of CHO^R^C5 and CHO^R^C5/Bcl-2 cells to tamoxifen (Fig. 4B).

### Oxidative stress mediates collateral sensitivity to tamoxifen

**Fig. 5:**
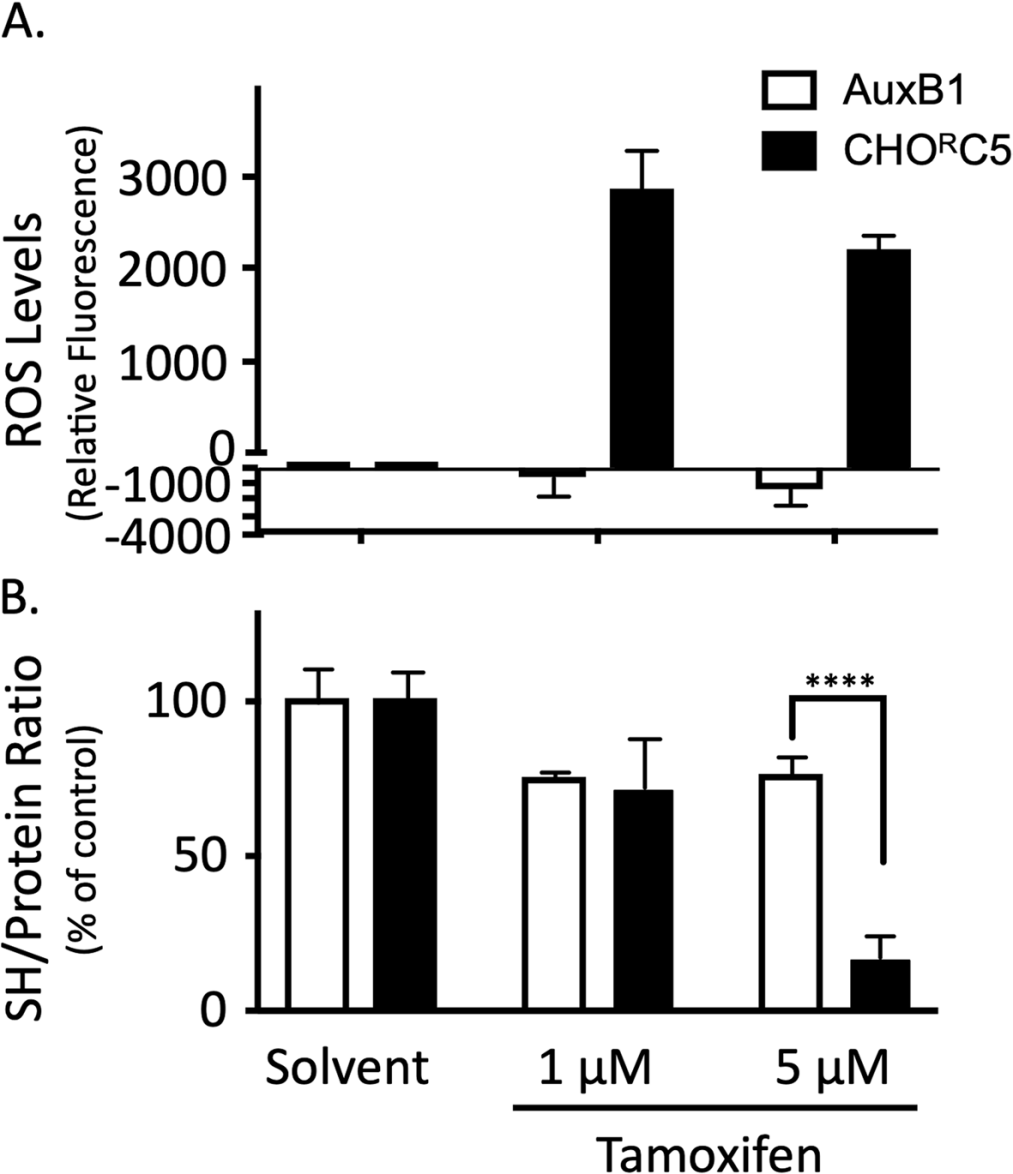
Tamoxifen treatment modulates the oxidative status of drug resistant cells – The effects of increasing concentrations of tamoxifen on the oxidative status of AuxB1 and CHO^R^C5 cells. Panel (**A**) and (**B**) shows drug sensitive (AuxB1) and -resistant (CHO^R^C5) cells incubated without and with tamoxifen (1 and 5 µM) for 24 h followed by a measure of total reduced cellular thiols levels and reactive oxygen species (ROS) in cells. Graphs represent the mean ± SD of three independent experiments done in triplicates. (****) indicates, *P* < 0.0005, statistically significant difference

Earlier reports have shown that heightened P-gp ATPase leads to selective decrease in cellular ATP level and a rise in reactive oxygen species (ROS), likely due to higher electron leak from mETC though enhanced oxidative phosphorylation [15, 16]. Tamoxifen has been shown by several investigators to increase P-gp ATPase at clinically relevant concentrations (< 5 μM [21, 36, 37]). To determine if the presence of low concentrations of tamoxifen causes a differential increase of ROS levels in CHO^R^C5 versus AuxB1, cells were exposed for 24 h to tamoxifen (1 μM and 5 μM) and intracellular ROS was quantified using 2',7'-dichlorodihydrofluorescein diacetate (H_2_DCFDA) dye [38]. The results in Fig. 5A show significantly higher ROS levels in CHO^R^C5 cells when incubated with tamoxifen (1—5 μM) relative to AuxB1 cells. Given the differential effect of tamoxifen on ROS levels in P-gp-expressing cells and the correlation between ROS and cellular thiols [39], we sought to measure total thiol levels of AuxB1 and CHO^R^C5 cells in the absence and presence of tamoxifen (1—5 μM). Figure 5B shows total reduced-thiol levels in untreated and tamoxifen treated cells, whereby tamoxifen treatment caused a drastic decline in total reduced-thiol levels in CHO^R^C5 cells relative to AuxB1 cells at 5 μM. Interestingly, tamoxifen at 1 μM produced a small drop in reduced-thiol levels in both cell lines (Fig. 5B), despite of the fact that large differential increase in ROS levels between AuxB1 and CHO^R^C5 cells was measured at 1 μM tamoxifen. Similar divergence between the increase in ROS levels and a decrease in reduced-thiol levels is also observed with MDA-MB-231 and MDA-Doxo^400^ cells. MDA-Doxo^400^ cells treated with 1 – 10 μM tamoxifen led to a significant differential rise in ROS levels at 1 and 5 μM tamoxifen, while higher tamoxifen concentrations (25 μM) were required to detect a differential drop in reduced-thiol levels in MDA-Doxo^400^ (supplemental Fig. 4). This divergence between the differential rise in ROS and drop in reduced-thiol levels between drug sensitive and resistance cells is not entirely clear; but maybe due to enhanced rebound in the production of reductive molecules or potential in cells following an initial rise in ROS [40].

**Fig. 6:**
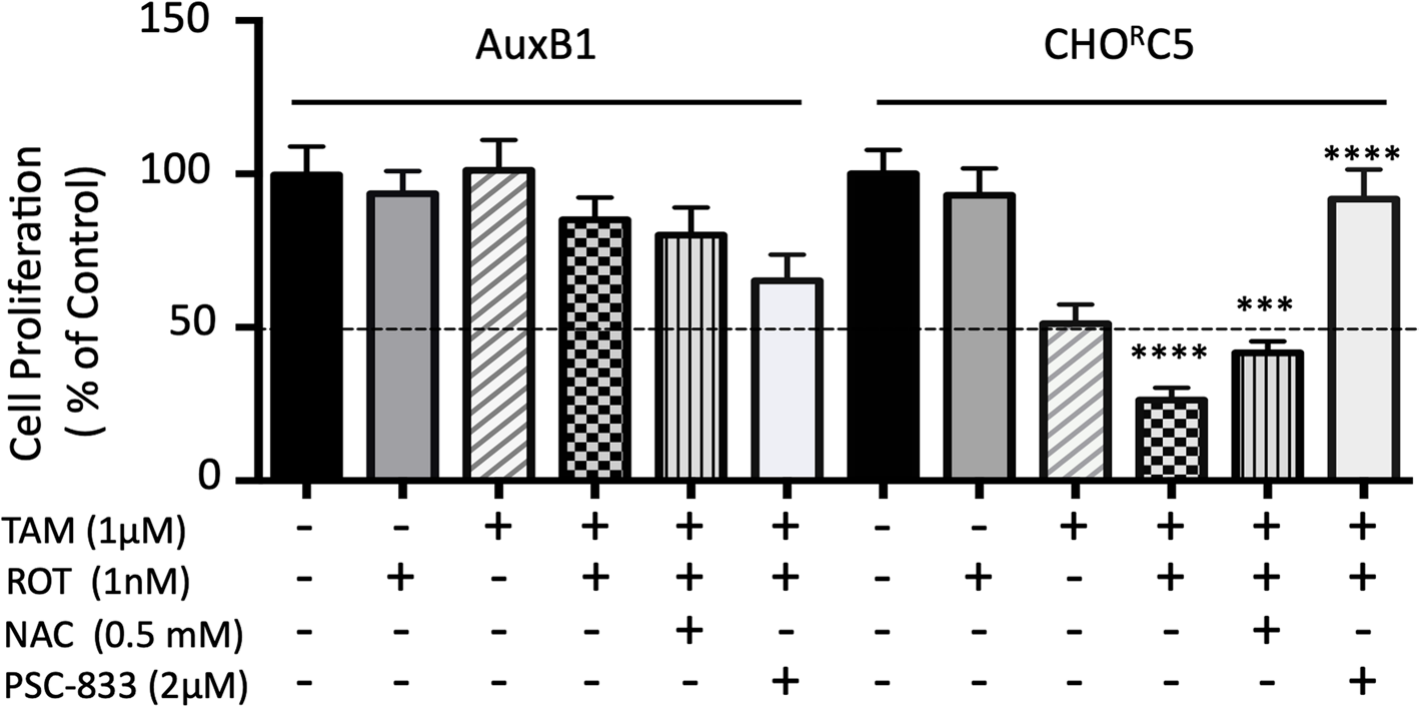
Pro- and anti-oxidants modulate collateral sensitivity to tamoxifen – The effects of rotenone (ROT; 1 nM), N-acetyl cysteine (NAC; 0.5 mM) and PSC-833 (2 µM) alone or combined with tamoxifen (1 µM) on the proliferation of AuxB1 and CHO^R^C5. The bar graphs represent the mean ± SD of three independent experiments done in triplicates. (****), (***) indicates, *P* < 0.0001, statistically significant difference

To decode tamoxifen-induced changes in cellular redox potential in treated cells, an in vitro cell proliferation assay was performed on AuxB1 and CHO^R^C5 cells treated with tamoxifen in combination with ROS generating drug (rotenone [41]), thiol regenerating agent (N-acetylcysteine (NAC) [42]) and P-gp-ATPase inhibitor (PSC-833 [33]). Figure 6 shows tamoxifen alone at 1 μM produced ~ 50% inhibition of CHO^R^C5 cell growth, while the same tamoxifen concentrations had no effect on growth of AuxB1. The addition of rotenone alone showed moderate growth inhibition effects on drug resistant (CHO^R^C5) cells, and a lesser effect on drug sensitive (AuxB1) cells. Interestingly, the presence of tamoxifen and rotenone together produced the largest growth inhibitory effects on CHO^R^C5 that were reversed with the addition of NAC (0.5 mM) which increases reduced-thiols levels or PSC-833 (2 μM), an inhibitor of P-gp-ATPase activity (Fig. 6). Similar results, as in Fig. 6, were seen with MDA-MB-231 and MDA-Doxo^400^ cells treated with 3 μM tamoxifen without and with 6.25 nM rotenone, 1 mM NAC or 2 μM PSC-833 (supplemental Fig. 5). Taken together the results demonstrate a differential rise in ROS induced in P-gp-expressing drug resistant cells, relative to their drug sensitive parental cells that can be reversed by exogenously augmenting the cells reduce-thiol levels (*i.e.*, addition of NAC) and inhibition of P-gp-ATPase activity (*i.e.*, PSC-833) or promoted with the exogenous addition of ROS generating drugs (*e.g.*, rotenone).

### Rotenone synergizes with tamoxifen

**Fig. 7:**
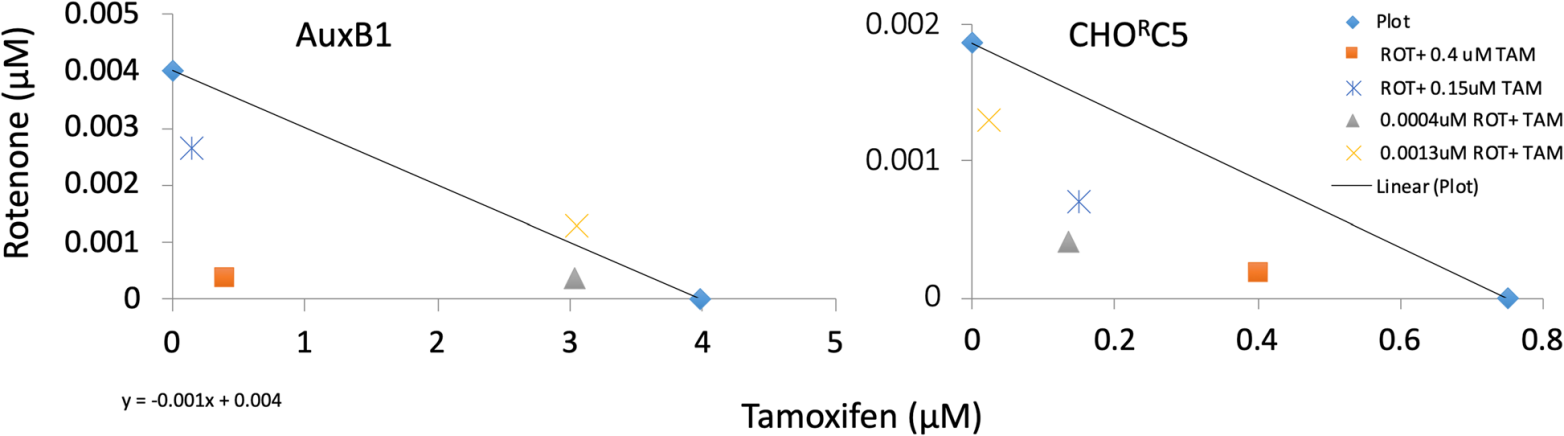
Rotenone and tamoxifen synergize to induce collateral sensitivity – Isobole analysis of interactions between tamoxifen and rotenone to induce collateral sensitivity in drug resistant cells (CHO^R^C5). Rotenone shows strong synergistic effect with 0.75 µM tamoxifen at 0.4 nM (Δ) and 1.3 nM (X) for CHO^R^C5. By contrast, at 0.4 nM (Δ) and 1.3 nM (X) rotenone concentrations, with 4 µM tamoxifen, AuxB1 shows moderate to antagonistic drug interactions

Based on the above results in Fig. 6, using tamoxifen and rotenone combination drugs, it was of interest to determine if rotenone synergizes with tamoxifen in their collateral sensitivity effects. To identify the nature of the interaction between tamoxifen and rotenone, fractional inhibitory concentrations (FIC) values derived from IC_50_ values were determined for each of the compound alone and in combination. Figure 7 shows the results of the FIC-based isobologram analysis, demonstrating a strong synergy for tamoxifen and rotenone for CHO^R^C5 with the mean FIC_index_ = 0.4 (FIC values below 0.5 are considered strong, while between 0.5 and 1 are considered moderate [30]). Synergy was also observed with AuxB1 cells exposed the drug combination (rotenone and tamoxifen), albeit at greater concentrations of these two drugs (Fig. 7). Similar synergy was observed for tamoxifen and rotenone with doxorubicin resistant triple negative breast cancer cells (MDA-Doxo^400^) by contrast to the parental drug sensitive cells (MDA-MB-231) (supplemental Fig. 6).

## Discussion

Tamoxifen, a non-steroidal anti-estrogen, has been used for decades in the treatment of estrogen-receptor positive breast cancer. Its anti-proliferative effect as transcription inhibitor of estrogen-responsive genes is well established [43]. However, tamoxifen has non-genomic effects that include the promotion of protein kinase C activity, intracellular calcium, mitochondrial stress, and stimulation of P-gp ATPase activity [24, 44, 45]. In this report we demonstrate for the first time the preferential targeting of P-gp-expressing MDR cells (CHO^R^C5 and MDA-Doxo^400^), relative to drug sensitive P-gp-negative cells (AuxB1 and MDA-MB-231), with clinically achievable concentrations of tamoxifen [21]. Moreover, the sensitivity of resistant cells (CHO^R^C5 and MDA-Doxo^400^) to tamoxifen correlated with P-gp expression level and reversed with specific inhibitor of P-gp ATPase (*e.g.*, PSC-833). Importantly, our results show that low levels of P-gp expression, as seen in MDA-Doxo^400^ cells (Fig. 2A), are sufficient to confer collateral sensitivity onto drug resistant cells. Together these results are consistent with earlier reports demonstrating a link between stimulation of P-gp ATPase and collateral sensitivity of drug resistant cells [15, 16]. In addition, we show that knockout of P-gp from drug resistant CHO^R^C5 (e.g., CHO^R^C5^ΔP−gp−A1^, and CHO^R^C5^ΔP−gp−A3^) cells completely reverses the collateral sensitivity of CHO^R^C5 cells to tamoxifen to the same level as AuxB1 or AuxB1^ΔP−gp^ cells. These results demonstrate the essential and direct role of P-gp in tamoxifen collateral sensitivity, while excluding the role of other cellular proteins.

The tamoxifen-induced mitochondrial stress has been attributed to its inhibition of complex I and III of the mitochondria electron transport complexes (mETC) [46–48] and a consequent increase of intracellular ROS in both ER-α + and ER-α- cells [49]. Consequently, the expected rise of ROS in ER-α- cells [49] is consistent with our findings in this study, as observed with MDA-MB-231 cells. However, tamoxifen shows significantly higher ROS levels in MDA-Doxo^400^, likely due to its stimulation of P-gp ATPase, beyond its effect on mETC. In support of the latter, it is noteworthy that the addition of rotenone, potent inhibitors of complex I of mETC [50], to CHO cells showed modest collateral sensitivity, significantly less than tamoxifen alone (supplemental Fig. 7). The modest increase in the sensitivity of CHO^R^C5 and MDA-Doxo^400^ cells to rotenone, relative to AuxB1 and MDA-MB-231, is likely due to the basal or unstimulated P-gp ATPase that was reversible with PSC-833 [16].

Tamoxifen and its metabolites have been shown to cause a concentration-dependent biphasic stimulation of P-gp ATPase (supplemental Fig. 8 [51],). This biphasic stimulation effect on P-gp ATPase activity has been observed with other drugs (*e.g.*, verapamil, progesterone, and deoxycorticosterone [15], whereby 1–5 μM of tamoxifen stimulate P-gp ATPase; while ≥ 10 μM of tamoxifen did not stimulate P-gp ATPase (supplemental Fig. 8 [51],). These results are consistent with the observed increase in ROS at lower concentrations of tamoxifen, as higher concentrations did not lead to higher ROS levels. Further support for tamoxifen-induced increase in ROS is provided by the decrease in total reduced thiol levels in P-gp-expressing cells and the reversal of its effect on cell growth in the presence of N-acetyl cysteine. Together these results suggest that P-gp overexpression confers tamoxifen induced oxidative cell death of drug resistant cells. A similar mechanism for the selective targeting of P-gp overexpressing cells was proposed for thiosemicarbazone derivative (*e.g.*, NSC73306) [18]. However, unlike tamoxifen, NSC73306 did not interact nor stimulated P-gp ATPase but is thought to act as redox cycling agent in the presence of metal ions [18]. Interestingly, NSC73306-induced collateral sensitivity was reversed by inhibitors of P-gp ATPase [19]. The latter observation suggest that P-gp-basal or unstimulated ATPase activity could be the trigger for oxidative stress above the redox equilibrium threshold. More recently, Al-Akra *et. al.* [52] have suggested that the thiosemicarbazone-mediated collateral sensitivity is likely due to the presence of external stress that leads to increased reactive oxygen species and internalization of P-gp which actively accumulates copper bound-thiosemicarbazone into lysosomal compartments leading to autophagic cell death. However, if tamoxifen-induced collateral sensitivity was mediated through increased autophagy; the addition of bafilomycin A1 (an inhibitor of V-type ATPase and an inhibitor of autophagy [53]) was expected to reverse tamoxifen induced collateral sensitivity. Our results (supplemental Fig. 9*)* shows that the presence of bafilomycin A1 (0.25 nM or 1 nM) did not have a significant effect on tamoxifen induced collateral sensitivity in CHO^R^C5 cells.

It is believed that chemotherapeutic treatment of cancer patients leads to the enrichment of resistant tumor cells which may express higher levels of P-gp [6]. Hence, it is tempting to speculate that tamoxifen efficacy in delaying the reoccurrence of breast cancer is due, in part, to its collateral sensitivity effect to selectively target P-gp overexpressing breast cancer cells. Interestingly, Tu *et. al.* [54] demonstrated that increased expression of enolase I in human breast cancer patients’ tumors correlate with increased resistance to tamoxifen. The latter results are consistent with our recent findings [55], whereby enolase I expression is inversely linked to P-gp expression, and drug resistant P-gp-expressing tumor cells escape tamoxifen sensitivity by down-regulating P-gp expression to decrease their ATP requirement to fuel P-gp function (e.g., ATP production through oxidative phosphorylation) with a consequent rise in enolase I function (ATP production through glycolysis). Hence, the findings that tamoxifen resistance is associated with increased enolase I expression is consistent with the premise that tamoxifen selectively targets P-gp-expressing tumor cells which have higher ATP needs to fuel P-gp. Consequently, the findings of this study may provide a rationale for increasing the efficacy of tamoxifen treatment through drug combinations that independently increase ROS and synergize to: a) better target P-gp expressing cells; b) reduce tamoxifen concentrations below clinically achievable levels; and c) reduce cellular toxicity of collateral sensitivity drugs due to off-target effects. Indeed, results in this study show rotenone, an inhibitor of complex I of the mETC, synergize with tamoxifen to increase the collateral sensitivity of CHO^R^C5 and MDA-Doxo^400^ drug resistant cells. Although it is currently not clear if tamoxifen is effective in targeting other types of drug resistant cells that over-express P-gp, based on our current understanding of the mechanism of collateral sensitivity of P-gp-overexpressing cells, we speculate that tamoxifen alone or in combination with rotenone could reduce the rise of drug resistant tumor cells. Taken together, the findings in this study point to the use of P-gp-induced ROS as a mechanism to limit the rise of drug efflux dependent resistant tumor cells pre- or post-treatment with chemotherapeutic drugs. Indeed, similar findings have been demonstrated with another ABC drug efflux mechanism, ABCC1 or MRP1 [56]. With respect to the latter, we have previously demonstrated that apigenin or verapamil, which stimulate ABCC1-mediated drug efflux [57], led to the selective oxidative cell death of multidrug resistant cells expressing ABCC1, in the absence of cytotoxic drugs, due to rapid efflux of intracellular glutathione and a rise in ROS levels [56].

## Supplementary Information

**Additional file 1. Supplemental Figure 1. **P-gp expression in total cell lysates: Western blot protein extracts from wild-type chinese hamster ovary cell (AuxB1), drug resistant selected cells (CHO^R^C5), P-gp-knockout drug sensitive cells AuxB1^ΔP−gp^) and P-gp-knockout CHO^R^C5 (CHO^R^C5^ΔP−gp−A1^, and CHO^R^C5^ΔP−gp−A3^) cells probed with P-gp-directed monoclonal antibody (C494 mAb) and anti-α-tubulin

**Additional file 2. Supplemental Figure 2. **Effects of 4-hydroxy-tamoxifen alone and with PCS-833 on the growth of AuxB1 and CHO^R^C5 cells- Cells were plated 24hrs prior to the addition of increasing concentrations of 4-hydroxy-tamoxifen alone and in the presence of an inhibitor of P-gp ATPase (PSC-833). Cells growth was allowed for 8 days and colonies stained methylene blue. Cell growth was plotted as percent growth relative to control cells treated with carrier solvent without or with 2 µM PSC-833. Graph represent the mean SD of three independent experiments done in triplicates.

**Additional file 3. Supplemental Figure 3. **Tamoxifen treatment of drug resistant breast cancer cells induces apoptosis- MDA-MB-231 and MDA-Doxo^400^ cells were incubated without and with 5 µM Tamoxifen and then stained with Hoechst dye for apoptotic cells, Photographs were taken at 2000X magnification (Nikon, Eclipse TE200, Quebec, Canada).

**Additional file 4. Supplemental Figure 4. **Tamoxifen treatment modulates the oxidative status of drug resistant cells-The effects of increasing concentrations of tamoxifen on the oxidative status of MDA-MB-231 and MDA-Doxo^400^ cells. Panel A and B shows drug sensitive (MDA-MB) and -resistant (MDA-Doxo^400^) cells incubated without and with tamoxifen (1, 5 and and 10 µM; Panel A and 10, 25 and 50 µM; Panel B) for 24hrs followed by a measure of reactive oxygen species (ROS; Panel A) and total reduced cellular thiols levels (Panel B) in cells. Graphs represent the mean ±SD of three independent experiments done in triplicates.

**Additional file 5. Supplemental Figure 5. **Pro- and anti-oxidants modulate collateral sensitivity to tamoxifen- The effects of rotenone (ROT; 6.25nM), N-acetylcysteine (NAC; 1 nM) and PSC-833 (2 µM) alone or combined with tamoxifen (3 µM) on the proliferation of MDA-MB-231 and MDA-Doxo^400^ cells. The bar graphs represent the mean ±SD of three independent experiment done in triplicates (**), (*) indicates, P<0.001, statistically significant difference.

**Additional file 6. Supplemental Figure 6. **Rotenone and tamoxifen synergize to induce collateral sensitivity - Isobole analysis of interactions between tamoxifen and rotenone to induce collateral sensitivity in drug resistant cells (MDA-Doxo^400^). Rotenone shows strong synergistic effect with 2.327 µM tamoxifen at 2 nM (□) for MDA-Doxo^400^ (Panel B). By contrast, MDA-MB-231 cells showed an antogonistic drug interaction 2 nM rotenone and 10.56 µM tamoxifen (Panel A).

**Additional file 7. Supplemental Figure 7. **Effects of rotenone on cell growth - Drug sensitive (AuxB1, MDA-MB-231) and -resistan (CHO^R^C5, MDA-Doxo^400^) cells were cultured in increasing concentration of rotenone for 8 days. Cell growth was plotted as percent of growth relative to cells treated with carrier solvent alone. Graphs represent the mean ± SD of two independent experiments done in triplicates.

**Additional file 8. Supplemental Figure 8.** Effects of Tamoxifen and Na-Orthovanadate on P-glycoprotein ATPase in AuxB1 and CHO^R^C5 cells. ATPase activity of AuxB1 and CHO^R^C5 was measure using purified plasma membranes expose to increasing concentrations of tamoxifen (0 - 1000 µM) alone, or in the present of Na-orthovanadate (500 µM).

**Additional file 9. Supplemental Figure 9. **Effects of Bafilomycin A and tamoxifen - Drug sensitive (AuxB1) and resistant (CHO^R^C5) cells were allowed to profilerate in the present of increasing concentrations of tamoxifen without and with Bafilomycin A1 (BfA; 0.25 and 1 nM). Cell proliferation was assessed as described in the methods section. Graphs represent the mean ± SD of three independent experiments done in triplicate.

**Additional file 10.**

**Additional file 11.**

## Data Availability

All data generated or analysed during this study are included in this published article and its supplementary information files.
