# Supplementary figures and images for: The anti-estrogen receptor drug, tamoxifen, is selectively Lethal to P-glycoprotein-expressing Multidrug resistant tumor cells
Source: BMC Cancer. 2023 Jan 6;23:24. doi: 10.1186/s12885-022-10474-x (PMC9824978; doi:10.1186/s12885-022-10474-x)

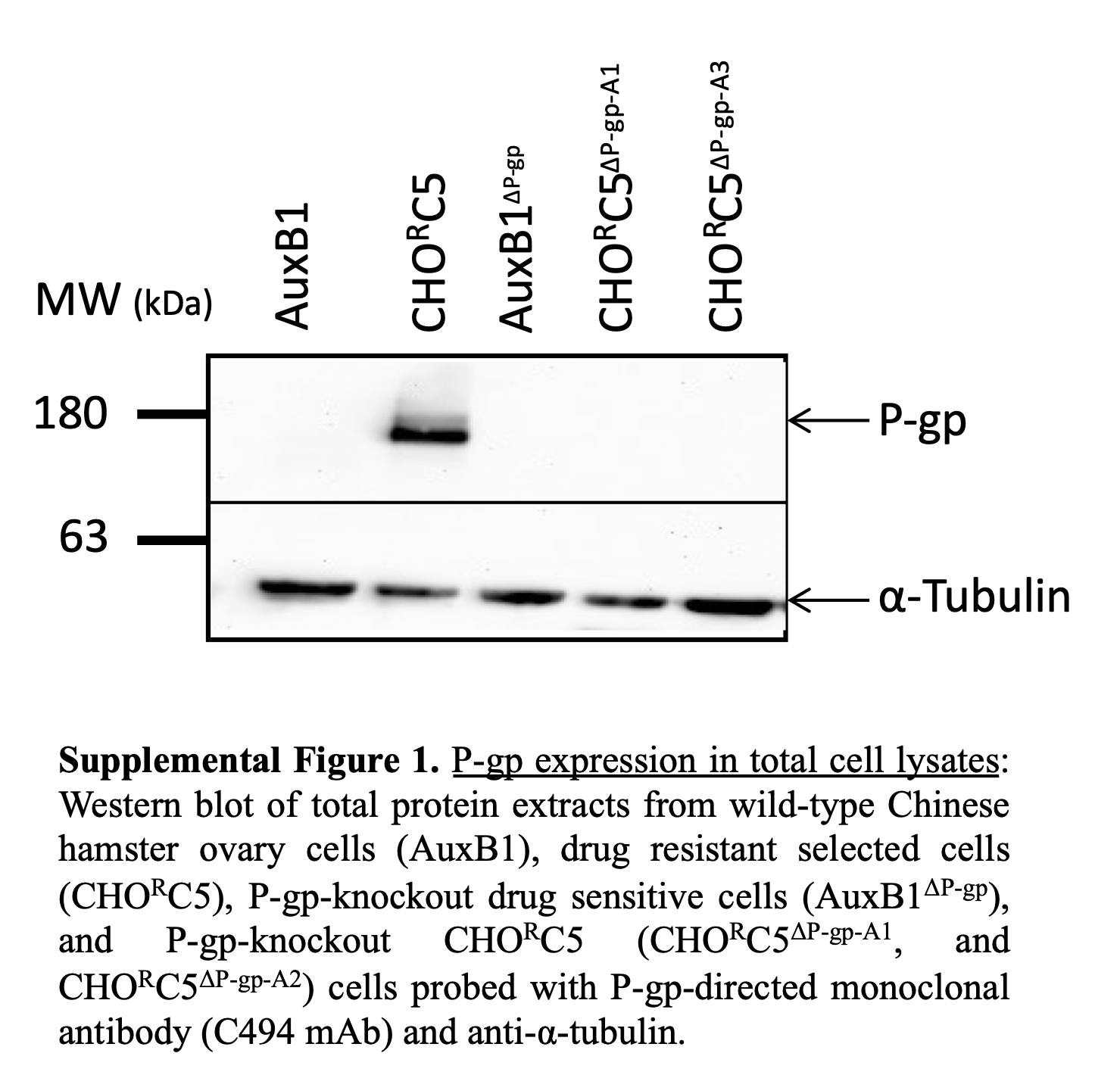

Supplement: Supplementary file 1 — Additional file 1. Supplemental Figure 1. P-gp expression in total cell lysates: Western blot protein extracts from wild-type chinese hamster ovary cell (AuxB1), drug resistant selected cells (CHORC5), P-gp-knockout drug sensitive cells AuxB1ΔP−gp) and P-gp-knockout CHORC5 (CHORC5ΔP−gp−A1, and CHORC5ΔP−gp−A3) cells probed with P-gp-directed monoclonal antibody (C494 mAb) and anti-α-tubulin [file 12885_2022_10474_MOESM1_ESM.tiff]

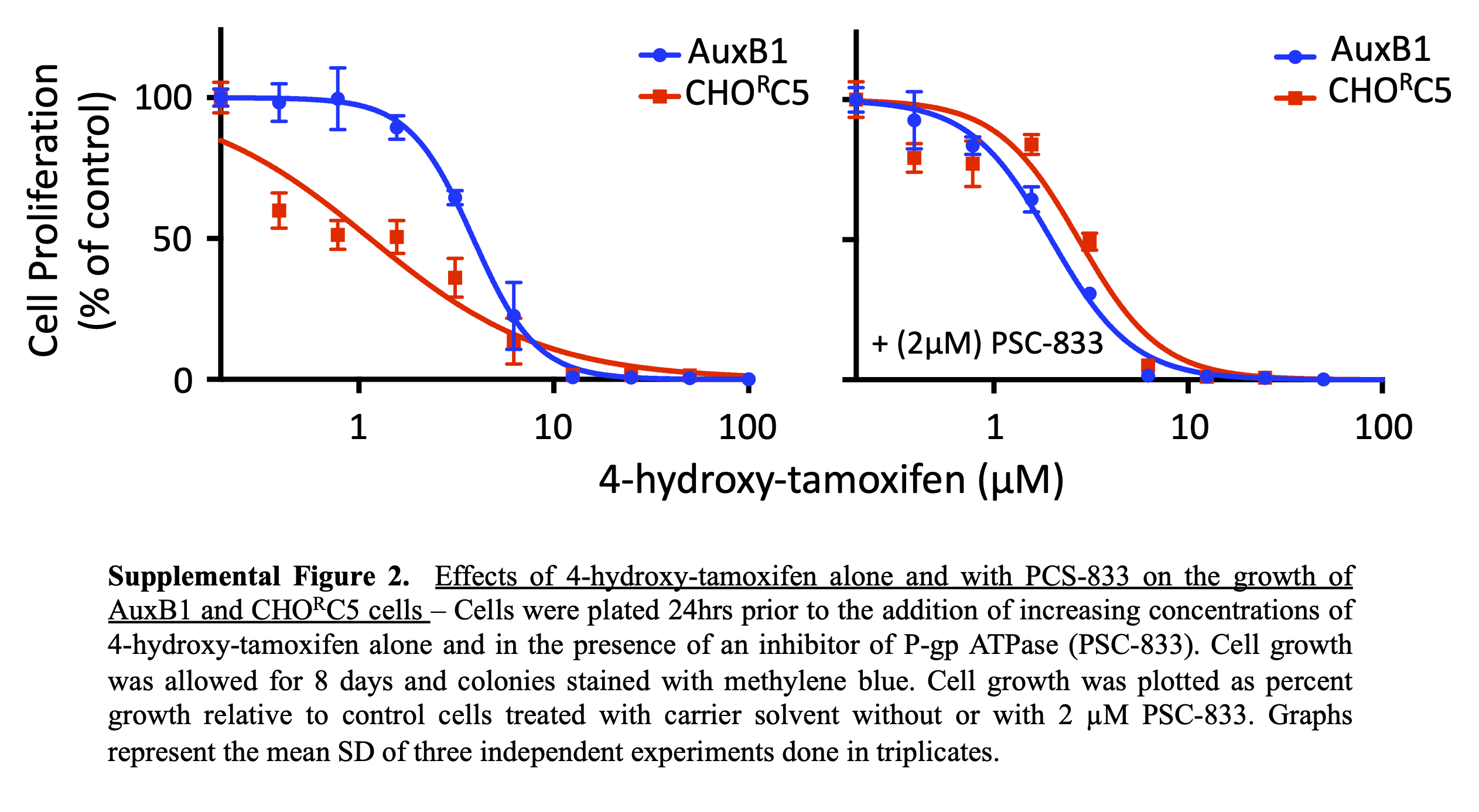

Supplement: Supplementary file 2 — Additional file 2. Supplemental Figure 2. Effects of 4-hydroxy-tamoxifen alone and with PCS-833 on the growth of AuxB1 and CHORC5 cells- Cells were plated 24hrs prior to the addition of increasing concentrations of 4-hydroxy-tamoxifen alone and in the presence of an inhibitor of P-gp ATPase (PSC-833). Cells growth was allowed for 8 days and colonies stained methylene blue. Cell growth was plotted as percent growth relative to control cells treated with carrier solvent without or with 2 µM PSC-833. Graph represent the mean SD of three independent experiments done in triplicates. [file 12885_2022_10474_MOESM2_ESM.tiff]

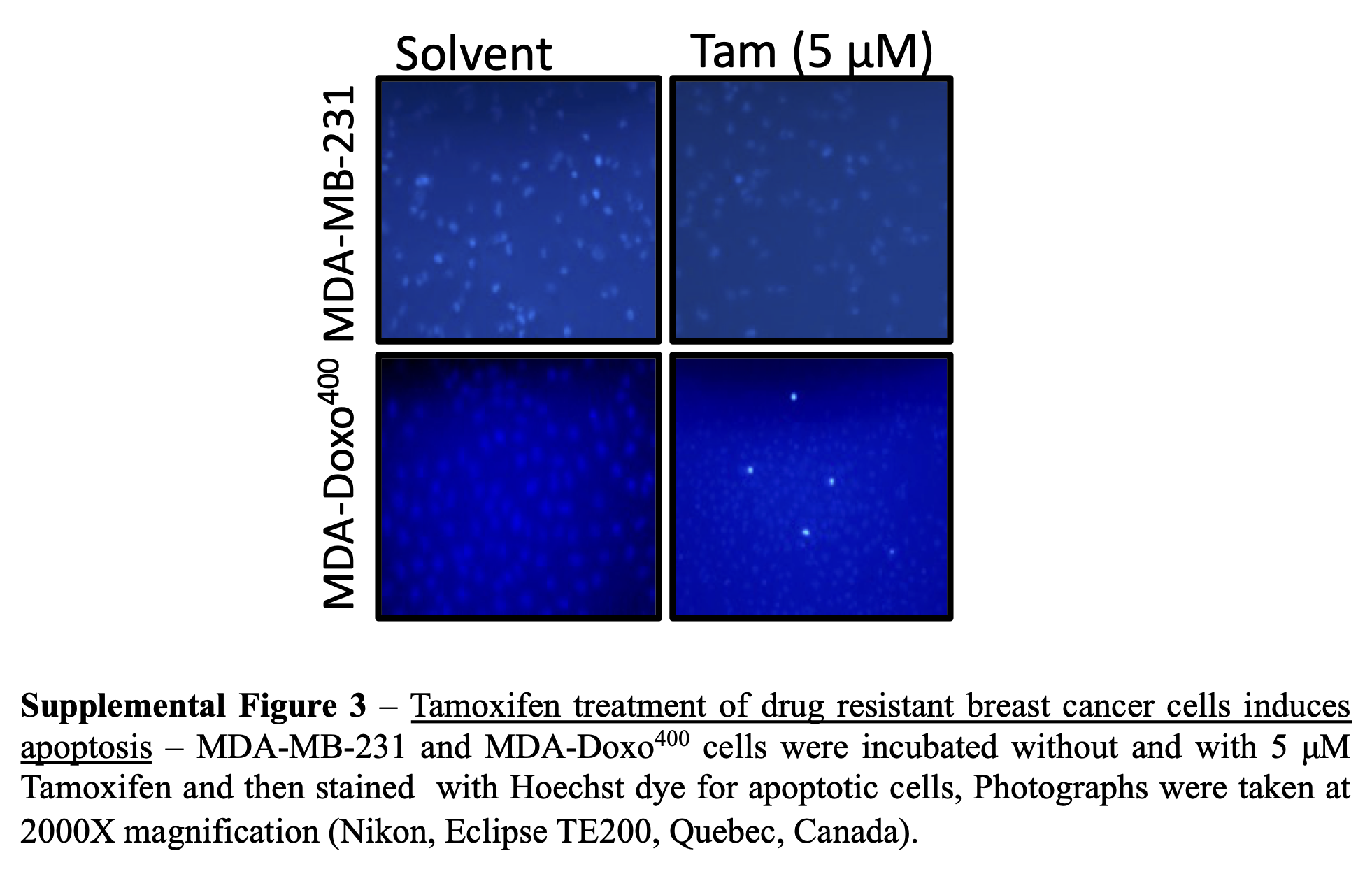

Supplement: Supplementary file 3 — Additional file 3. Supplemental Figure 3. Tamoxifen treatment of drug resistant breast cancer cells induces apoptosis- MDA-MB-231 and MDA-Doxo400 cells were incubated without and with 5 µM Tamoxifen and then stained with Hoechst dye for apoptotic cells, Photographs were taken at 2000X magnification (Nikon, Eclipse TE200, Quebec, Canada). [file 12885_2022_10474_MOESM3_ESM.tiff]

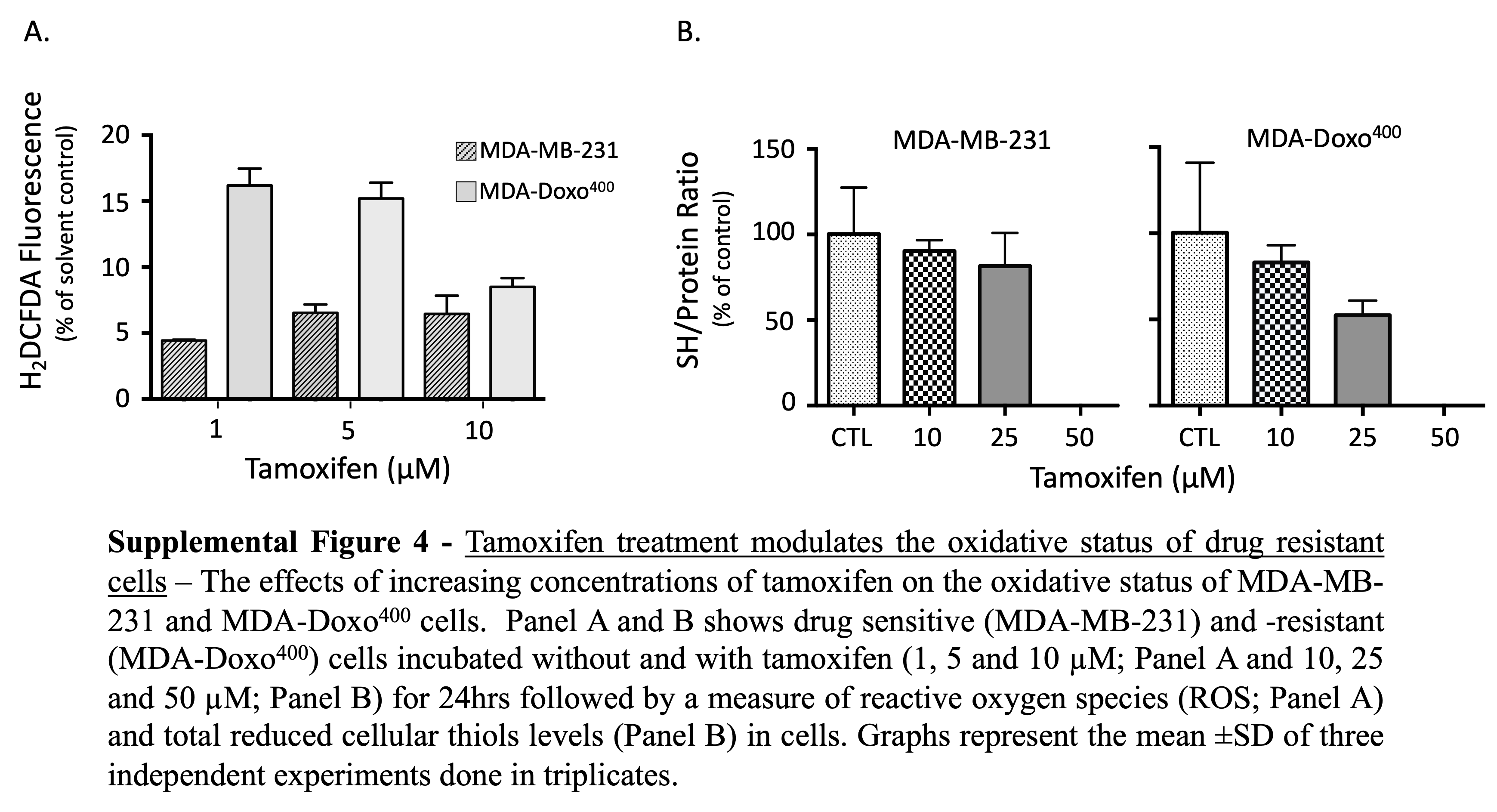

Supplement: Supplementary file 4 — Additional file 4. Supplemental Figure 4. Tamoxifen treatment modulates the oxidative status of drug resistant cells-The effects of increasing concentrations of tamoxifen on the oxidative status of MDA-MB-231 and MDA-Doxo400 cells. Panel A and B shows drug sensitive (MDA-MB) and -resistant (MDA-Doxo400) cells incubated without and with tamoxifen (1, 5 and and 10 µM; Panel A and 10, 25 and 50 µM; Panel B) for 24hrs followed by a measure of reactive oxygen species (ROS; Panel A) and total reduced cellular thiols levels (Panel B) in cells. Graphs represent the mean ±SD of three independent experiments done in triplicates. [file 12885_2022_10474_MOESM4_ESM.tiff]

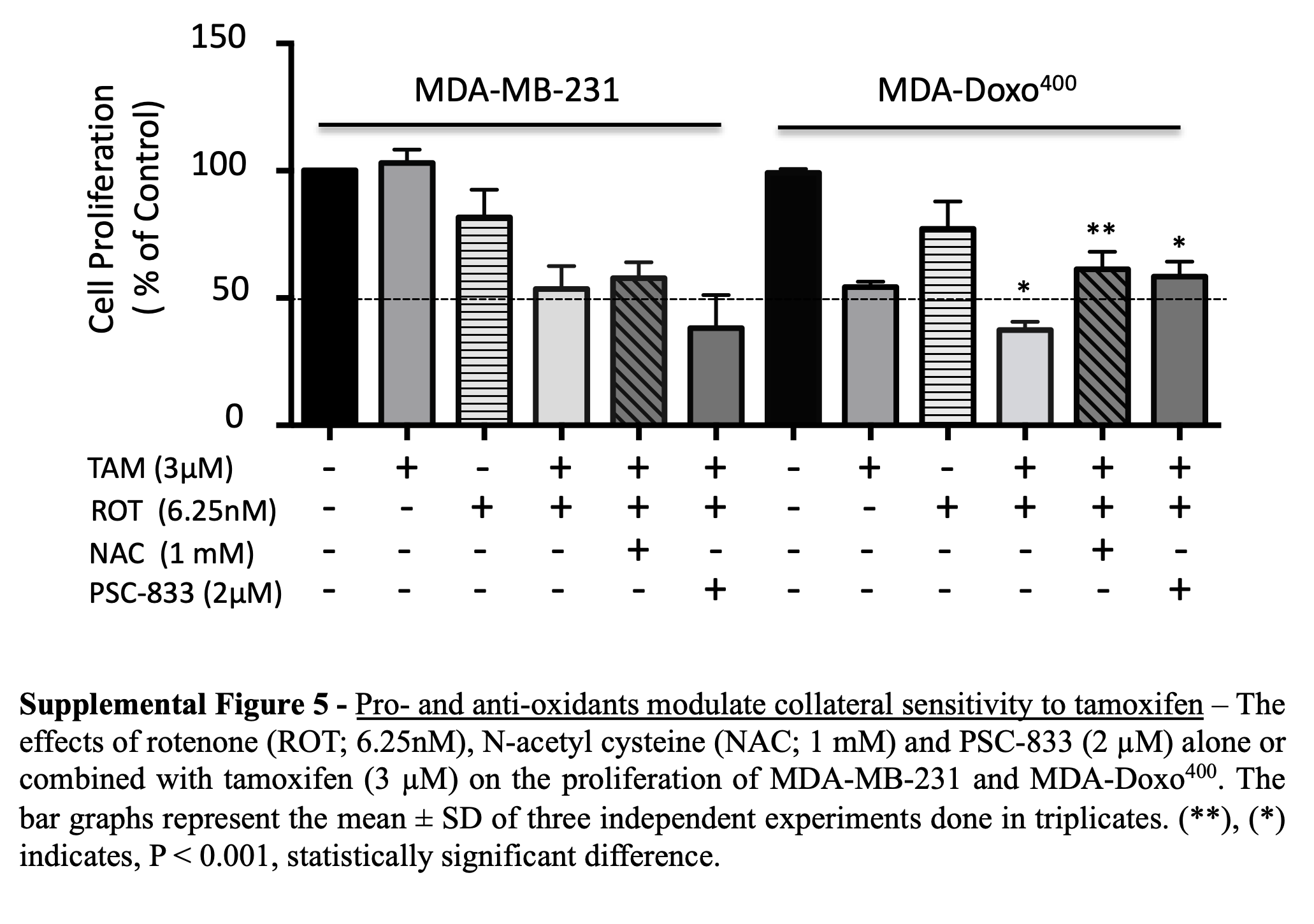

Supplement: Supplementary file 5 — Additional file 5. Supplemental Figure 5. Pro- and anti-oxidants modulate collateral sensitivity to tamoxifen- The effects of rotenone (ROT; 6.25nM), N-acetylcysteine (NAC; 1 nM) and PSC-833 (2 µM) alone or combined with tamoxifen (3 µM) on the proliferation of MDA-MB-231 and MDA-Doxo400 cells. The bar graphs represent the mean ±SD of three independent experiment done in triplicates (**), (*) indicates, P<0.001, statistically significant difference. [file 12885_2022_10474_MOESM5_ESM.tiff]

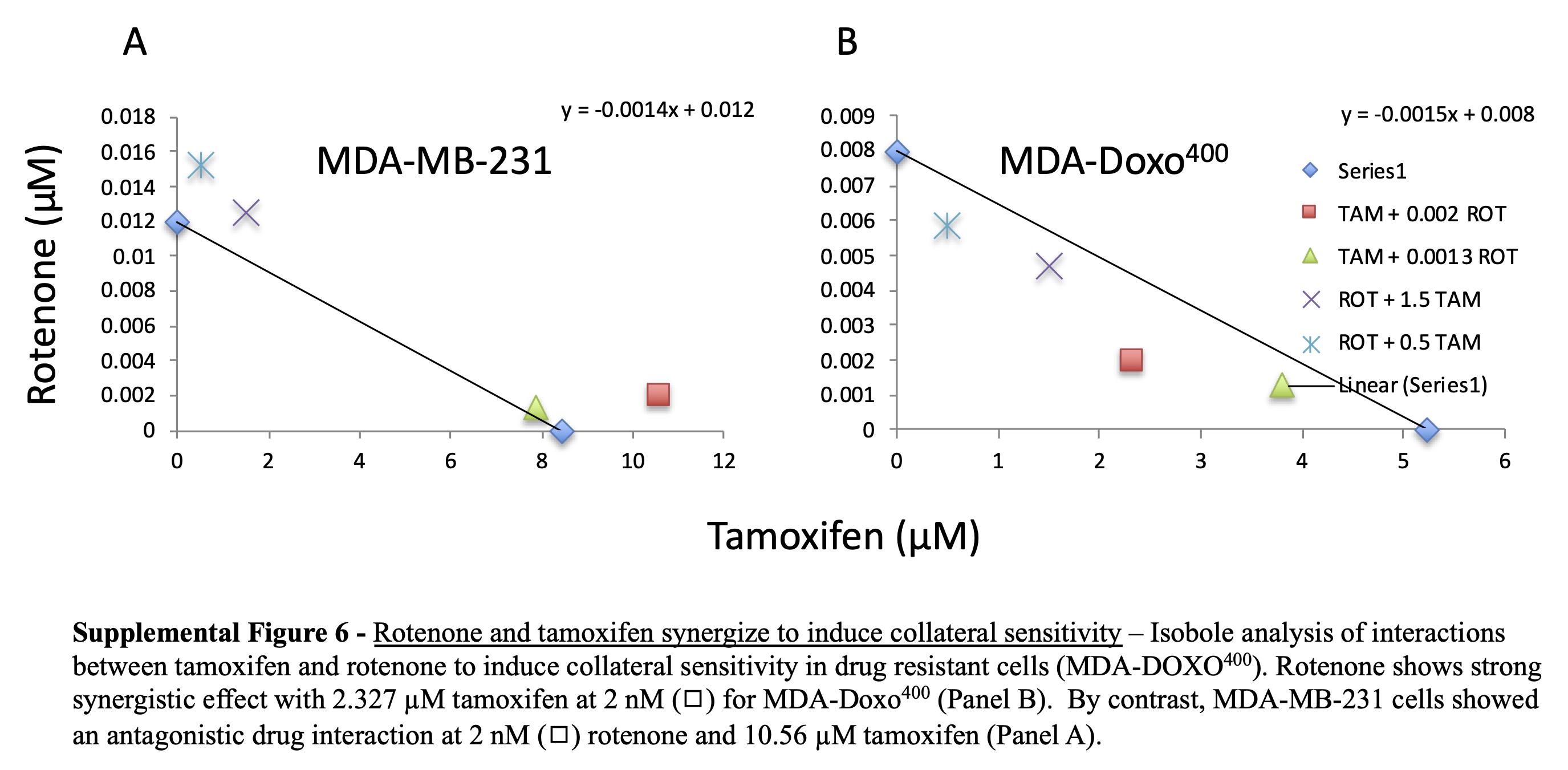

Supplement: Supplementary file 6 — Additional file 6. Supplemental Figure 6. Rotenone and tamoxifen synergize to induce collateral sensitivity - Isobole analysis of interactions between tamoxifen and rotenone to induce collateral sensitivity in drug resistant cells (MDA-Doxo400). Rotenone shows strong synergistic effect with 2.327 µM tamoxifen at 2 nM (□) for MDA-Doxo400 (Panel B). By contrast, MDA-MB-231 cells showed an antogonistic drug interaction 2 nM rotenone and 10.56 µM tamoxifen (Panel A). [file 12885_2022_10474_MOESM6_ESM.tiff]

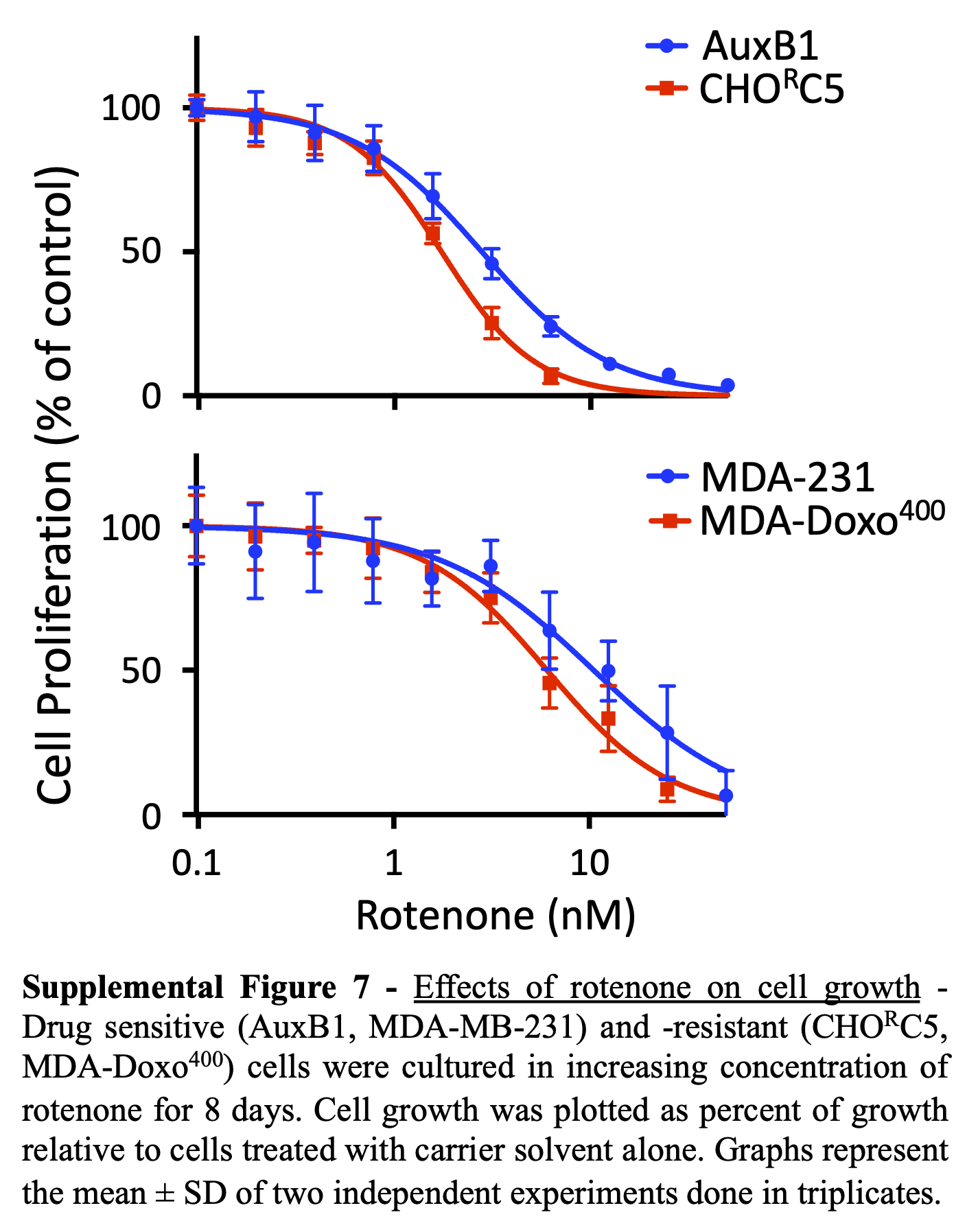

Supplement: Supplementary file 7 — Additional file 7. Supplemental Figure 7. Effects of rotenone on cell growth - Drug sensitive (AuxB1, MDA-MB-231) and -resistan (CHORC5, MDA-Doxo400) cells were cultured in increasing concentration of rotenone for 8 days. Cell growth was plotted as percent of growth relative to cells treated with carrier solvent alone. Graphs represent the mean ± SD of two independent experiments done in triplicates. [file 12885_2022_10474_MOESM7_ESM.tiff]

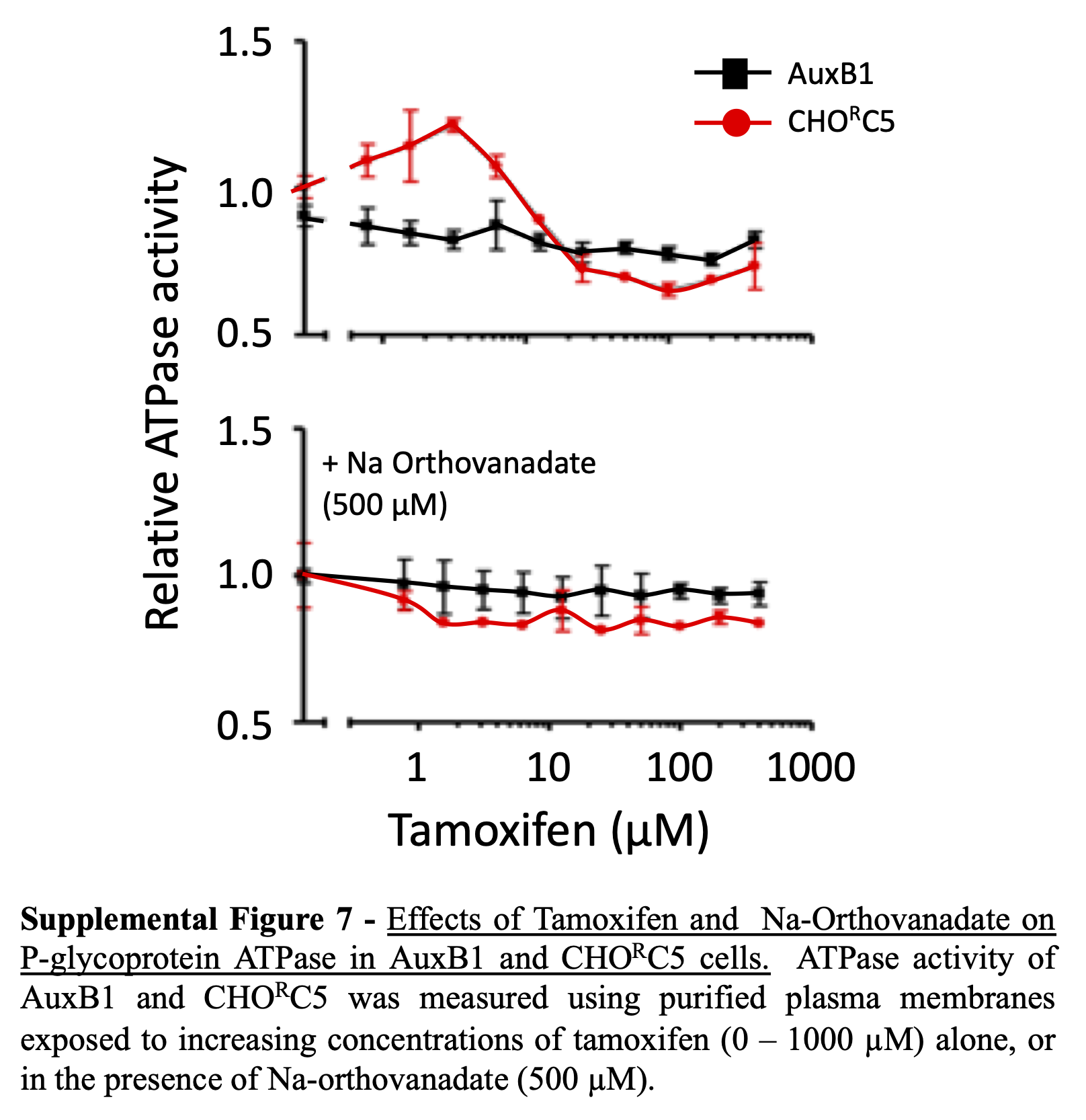

Supplement: Supplementary file 8 — Additional file 8. Supplemental Figure 8. Effects of Tamoxifen and Na-Orthovanadate on P-glycoprotein ATPase in AuxB1 and CHORC5 cells. ATPase activity of AuxB1 and CHORC5 was measure using purified plasma membranes expose to increasing concentrations of tamoxifen (0 - 1000 µM) alone, or in the present of Na-orthovanadate (500 µM). [file 12885_2022_10474_MOESM8_ESM.tiff]

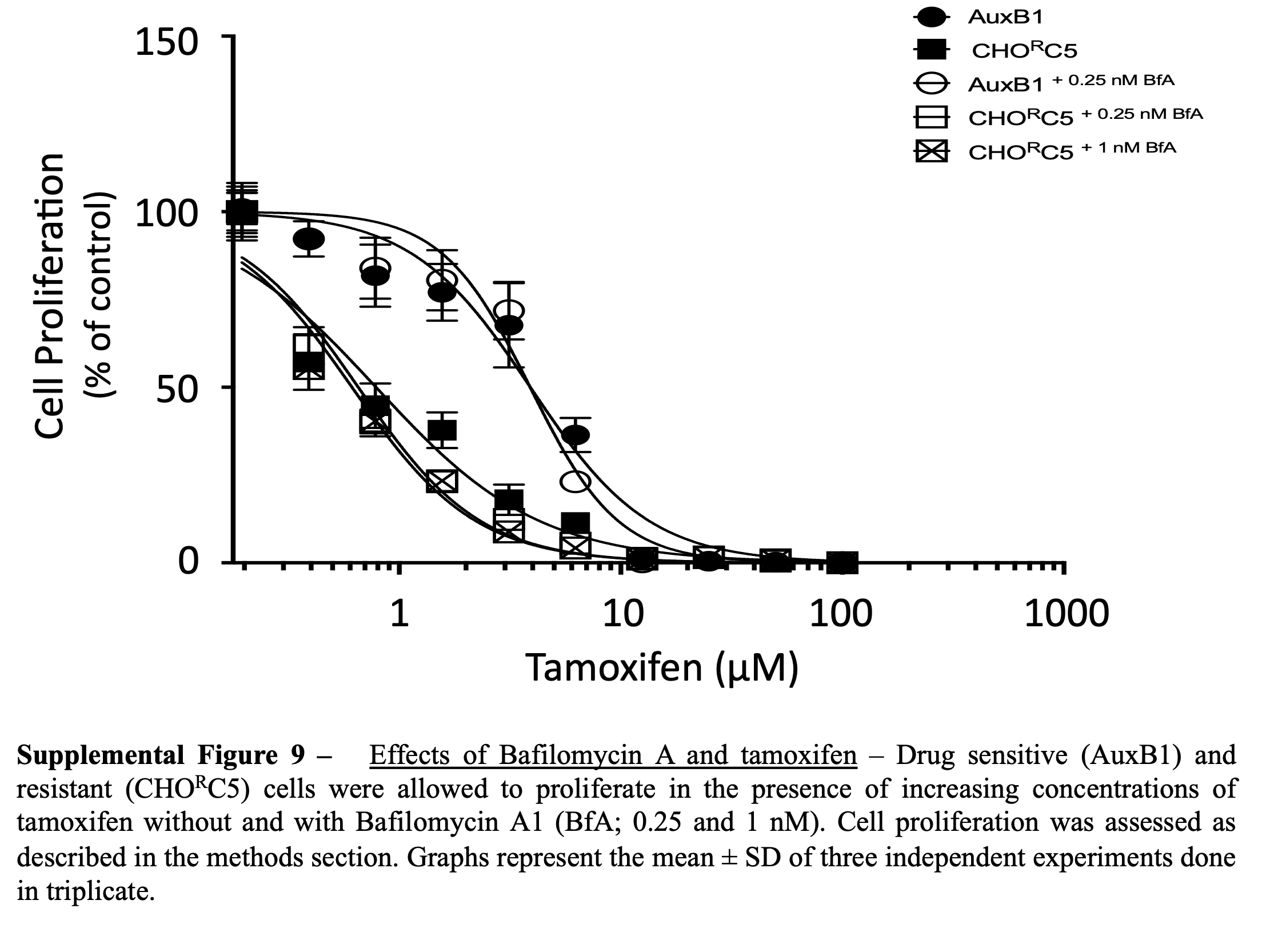

Supplement: Supplementary file 9 — Additional file 9. Supplemental Figure 9. Effects of Bafilomycin A and tamoxifen - Drug sensitive (AuxB1) and resistant (CHORC5) cells were allowed to profilerate in the present of increasing concentrations of tamoxifen without and with Bafilomycin A1 (BfA; 0.25 and 1 nM). Cell proliferation was assessed as described in the methods section. Graphs represent the mean ± SD of three independent experiments done in triplicate. [file 12885_2022_10474_MOESM9_ESM.tiff]

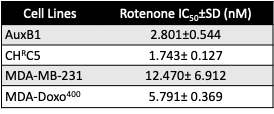


**Table I.** Effects of rotenone alone on drug-sensitive and –resistant CHO or MDA cells

Supplement: Supplementary file 11 — Additional file 11. [file 12885_2022_10474_MOESM11_ESM.docx]
