# Supplementary material for: The anti-estrogen receptor drug, tamoxifen, is selectively Lethal to P-glycoprotein-expressing Multidrug resistant tumor cells
Source: BMC Cancer. 2023 Jan 6;23:24. doi: 10.1186/s12885-022-10474-x (PMC9824978; doi:10.1186/s12885-022-10474-x)
Supplement: Supplementary file 10 — Additional file 10. [file 12885_2022_10474_MOESM10_ESM.docx]

*SUPPLEMENTAL METHODS*

*Measurements of ATP Pools* **-** Intracellular ATP levels were determined by a chemo-luminescence method utilizing luciferase. Cells (AuxB1 and CHO^R^C5) were plated at 1 × 10^4^ to 2.5 × 10^4^, in black 96 well plates and incubated for 24 h at 37 °C, without and with increasing concentrations of tamoxifen (1-10 μM) plus minus P-gp-ATPase inhibitor PSC833 (2 μM). Cells were washed twice with PBS and total cellular ATP level was measured with ATPLite (PerkinElmer) according to manufacturer's instruction. Plates were dark adapted for 10 min prior to reading the luminescence on BMG Fluorometer (FluoroStar Galaxy, Germany) with gain value of 100.

*Plasma membrane and ATPase assay -* Plasma membranes from AuxB1 and CHO^R^C5 cells were prepared as previously described (*1*). P-gp ATPase activity was measured in a reaction mix containing increasing concentrations of tamoxifen (1-100 μM) without or with 500 μM Na-orthovanadate and plasma membranes in ice-cold Buffer I (100 mM Tris-HCl, pH 8.0, 4 mM DTT, 100 mM KCl, 10 mM MgCl_2_, 10 mM NaN_3_, 4 mM EGTA, 2 mM Ouabain and 3 mM ATP). Membranes from AuxB1 and CHO^R^C5 cells were added to the wells (30 μl at 1 μg/well) and incubated at 37 °C for 30 min. Tamoxifen alone or together with Na-orthovanadate was added to the wells (10 μl/well) and plates were incubated for one hour at 37 °C. The reaction was stopped with the addition of 200 μl of buffer S containing [0.2% (w/v) ammonium molybdate, 1.3% (v/v) sulfuric acid, 0.9% (w/v) SDS, 2.3% (w/v) TCA and 1% (w/v) L-ascorbic acid]. Plates were incubated at room temperature for 75 min and absorbance read at 620 nm.

(1) Karwatsky, J., Lincoln, M.C., and Georges, E. (2003) A mechanism for P-glycoprotein-mediated apoptosis as revealed by verapamil hypersensitivity. *Biochemistry*. **42**, 12163-12173
